# Oligosilanylated Silocanes †

**DOI:** 10.3390/molecules26010244

**Published:** 2021-01-05

**Authors:** Mohammad Aghazadeh Meshgi, Alexander Pöcheim, Judith Baumgartner, Viatcheslav V. Jouikov, Christoph Marschner

**Affiliations:** 1 Institut für Anorganische Chemie, Technische Universität Graz, Stremayrgasse 9, A-8010 Graz, Austria; agmineralag@yahoo.com (M.A.M.); alexander.poecheim@tugraz.at (A.P.); 2 UMR 6226—ISCR, Université de Rennes 1, 35042 Rennes, France

**Keywords:** silanides, hypercoordination, cyclic voltammetry, DFT calculations

## Abstract

A number of mono- and dioligosilanylated silocanes were prepared. Compounds included silocanes with 1-methyl-1-tris(trimethylsilyl)silyl, 1,1-bis[tris(trimethylsilyl)silyl], and 1,1-bis[tris(trimethylsilyl)germyl] substitution pattern as well as two examples where the silocane silicon atom is part of a cyclosilane or oxacyclosilane ring. The mono-tris(trimethylsilyl)silylated compound could be converted to the respective silocanylbis(trimethylsilyl)silanides by reaction with KO*^t^*Bu and in similar reactions the cyclosilanes were transformed to oligosilane-1,3-diides. However, the reaction of the 1,1-bis[tris(trimethylsilyl)silylated] silocane with two equivalents of KO*^t^*Bu leads to the replacement of one tris(trimethylsilyl)silyl unit with a *tert*-butoxy substituent followed by silanide formation via KO*^t^*Bu attack at one of the SiMe_3_ units of remaining tris(trimethylsilyl)silyl group. For none of the silylated silocanes, signs of hypercoordinative interaction between the nitrogen and silicon silocane atoms were detected either in the solid state. by single crystal XRD analysis, nor in solution by ^29^Si-NMR spectroscopy. This was further confirmed by cyclic voltammetry and a DFT study, which demonstrated that the N-Si distance in silocanes is not only dependent on the energy of a potential N-Si interaction, but also on steric factors and through-space interactions of the neighboring groups at Si and N, imposing the orientation of the p_z_(N) orbital relative to the N-Si-X axis.

## 1. Introduction

Compared to carbon, for heavier group 14 elements hypercoordination is a fairly frequently observed phenomenon. Two major reasons are responsible for this. First, the heavier elements are more electropositive than carbon and when attached to strongly electronegative elements like oxygen, accumulate partial positive charges, which act as Lewis acidic electron acceptor sites. And second, these elements also have larger covalent radii and therefore penta- or even hexacoordination does not cause excessive steric strain. Nowadays, a number of compound classes featuring hypercoordination are well-investigated. Especially, the cage-type atrane molecules of heavier group 14 elements have received much attention [1,2] and recently we have contributed to these investigations by some studies of oligosilanyl-substituted silatranes [3,4,5,6,7].

One emerging interesting application of atrane-type molecules seems to be their use in cross-coupling reactions as a transfer vehicle [8,9,10,11,12,13].

## 2. Results

**Scheme 1:**
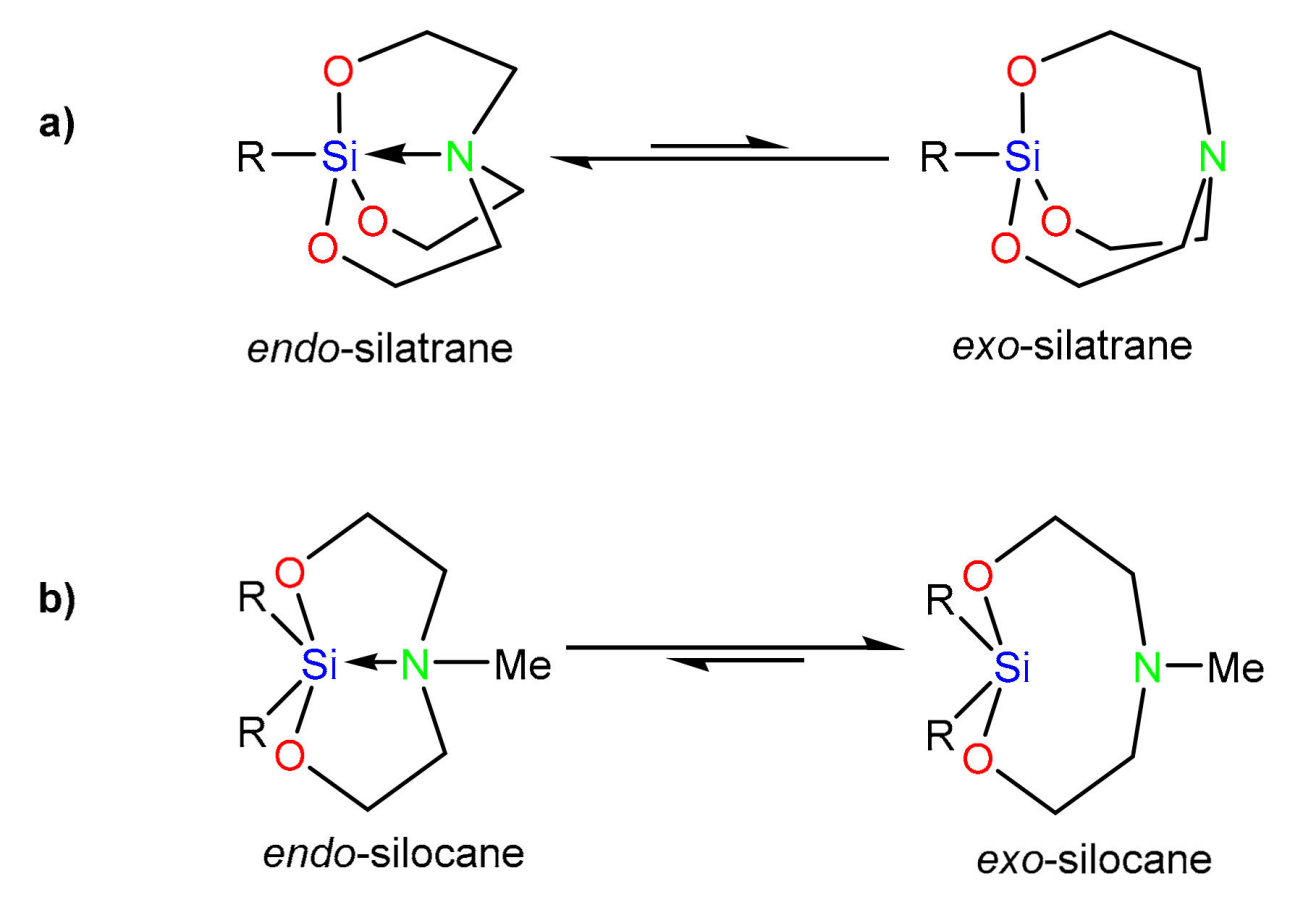
For silatranes (**a**) the *endo*-form and for silocanes (**b**) the *exo*-form is favored.

Silocanes are another member of the family of silanes with the possibility of internal hypercoordination. They are structurally related to the above-mentioned silatranes, but lack one bridging N-ethoxy arm, which allows for much more conformational freedom compared to the cage-type silatranes. Both compound classes nevertheless can engage in N→Si interaction to form hypercoordinated silicon compounds. In contrast to silatranes, the noncoordinating *exo*-form of silocanes is favored in most cases over the *endo*-form (Scheme 1) [3,4,14,15]. The reasons for this can be either steric (f.i., no structurally characterized N-Ph silocanes [16,17,18,19,20] with pronounced N→Si interaction are known, whereas a fair number of *exo* N-Me silocanes [6,20,21,22,23,24] are known) or electronic (at least one additional electronegative substituent seems to be required on the silocane Si to exhibit enough electrophilicity for a stable N→Si interaction).

Calculations have shown that the diminished transannular Si-N interaction of silocanes makes them more susceptible for hydrolysis of the Si-O bonds [25]. 

### 2.1. Synthesis

Our entry into the class of oligosilanylated silocanes initially occurred in the course of designing silyl ligands for coordination to lanthanide ions [6]. Silocanes can be regarded as structural variations of silatranes, where one of the bridges between the donor nitrogen and the acceptor silicon is removed. By this, the rigid bicyclic cage with enforced proximity of nitrogen and silicon is transformed into a 1-sila-2,8-dioxa-5-aminocyclooctane with a diminished propensity for transannular Si-N donor–acceptor interaction.

**Scheme 2:**
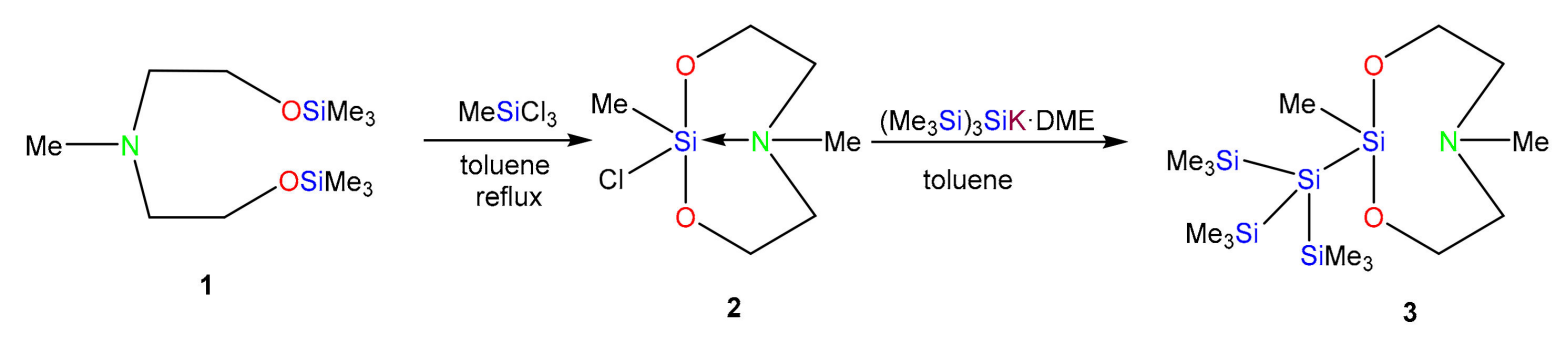
Preparation of oligosilanylated silocane compound **3**.

Reaction of trimethylsilylated methyldiethanolamine **1** with silanes carrying at least two leaving groups constitutes a straightforward way to the silocane core with a potential for further functionalization. Reaction of **1** with methyltrichlorosilane thus gave silocane **2**, which upon treatment with potassium tris(trimethylsilyl)silanide was converted to the oligosilanylated silocane **3** (Scheme 2) [6].

**Scheme 3:**
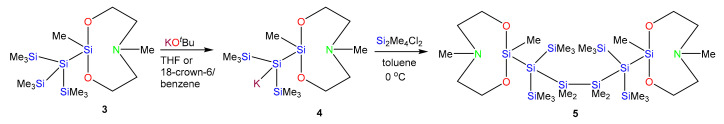
Formation of silocanyl-substituted bis(trimethylsilyl)silanide **4** and its conversion to the 1,4-disilocanylated tetrasilane **5.**

Synthesis of potassium silocanylbis(trimethylsilyl)silanide **4** was achieved subsequently by reaction of **3** with one equivalent of KO*^t^*Bu either in THF or in the presence of 18-crown-6 in benzene (Scheme 3). Attack of the alkoxide occurs selectively at one of the trimethylsilyl groups [6]. Synthesis of bis(oligosilanylsilocane) **5** was achieved by reaction of two equivalents of **4** with 1,2-dichlorotetramethyldisilane (Scheme 3) [26].

**Figure 1:**
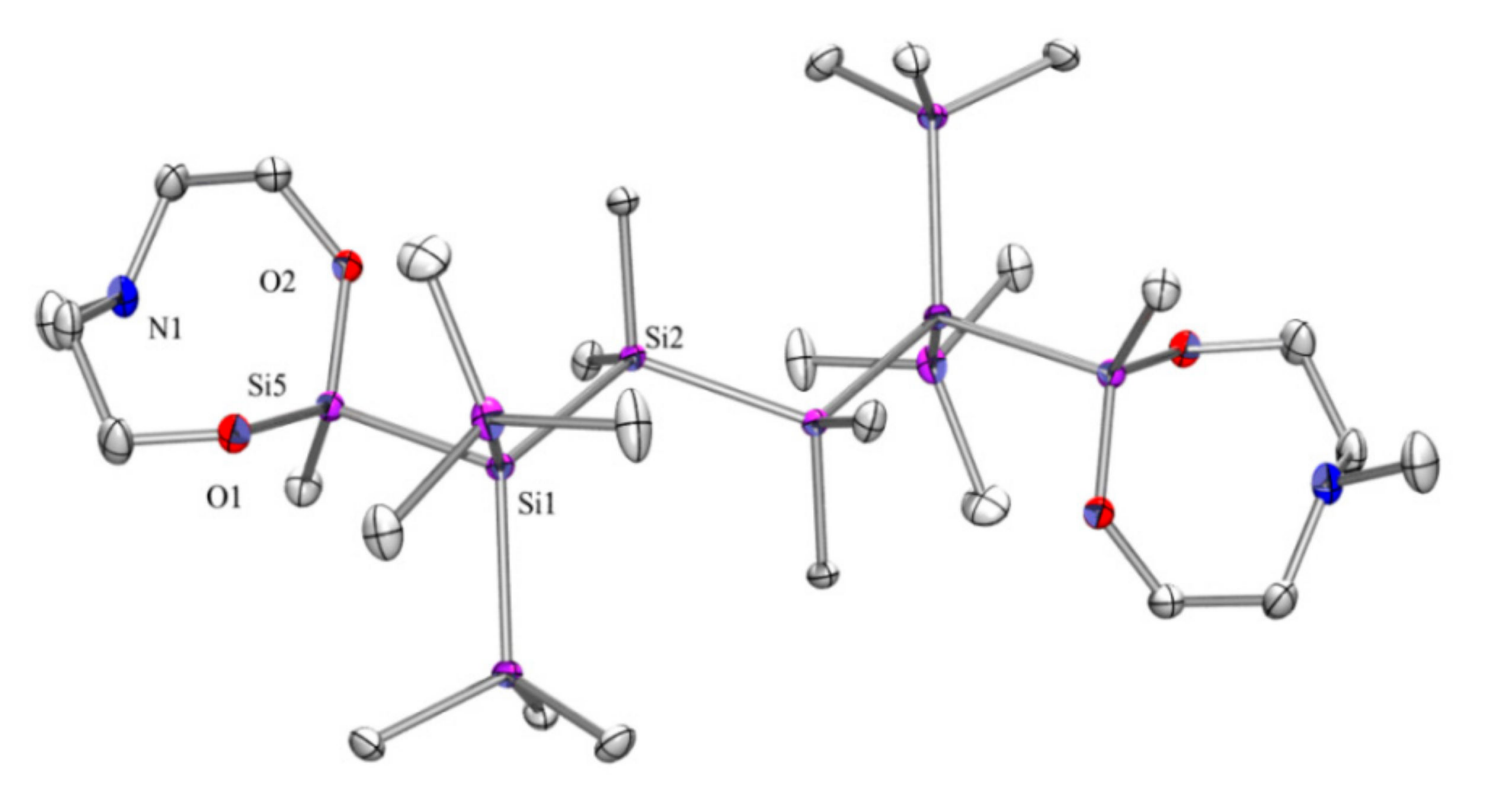
Molecular structure of **5** (thermal ellipsoid plot drawn at the 30% probability level). All hydrogen atoms are omitted for clarity (bond lengths in Å, angles in deg). Si(1)-Si(3) 2.3451(9), Si(1)-Si(5) 2.3564(9), Si(1)-Si(2) 2.3643(9), Si(2)-C(1) 1.885(3), Si(5)-O(2) 1.6456(17), Si(5)-O(1) 1.6474(17), N(1)-C(14) 1.440(4), O(1)-C(10) 1.416(3), Si(4)-Si(1)-Si(3) 108.61(4), O(2)-Si(5)-O(1) 113.50(10), O(1)-Si(5)-Si(1) 104.26(7).

The crystal structure of oligosilanylsilocane **5** (Figure 1) shows the large oligosilanyl substituent in the axial (N-Si-Si angle = 167.91(5) deg) and the smaller methyl group in the equatorial (N-Si-Me angle = 80.7(1) deg) position. The N-methyl group is oriented approximately parallel to the Si-methyl group. The compound features a Si-SiO_2_ bond length of 2.3564(9) Å, which is close to the Si-SiO_3_ bond length of our previously reported 1,2-tetramethyldisilanylene bridged bis(oligosilanylsilatrane) with respective Si-SiO_3_ bond lengths of 2.3504 Å and 2.3417 Å [4]. However, the Si-N distance of 2.934(2) Å in **5** is much longer than the respective Si-N distances of the respective oligosilanylene-bridged disilatrane with distances of 2.223 and 2.208 Å. While there is an evident N→Si interaction in **2** (Si-N distance 2.117(1) Å) caused by the electronegativity of the chlorine, the more-electron-donating oligosilanyl group renders the silocane silicon atom less electrophilic. Another difference between disilocane **5** and the analogous disilatrane is a shortening of the Si-O bonds with an average length of 1.64 Å in comparison to the Si-O bonds of the disilatrane with average lengths of 1.66 Å. A possible transannular N→Si interaction strongly depends on the substituents on silicon and nitrogen and in general it is less strong in silocanes than in silatranes. Considering the Si-N, Si-SiO_2_, and Si-SiO_3_ distances in the silocanyl and silatranyl groups of the oligosilanyl bridged compounds, it can be concluded that the N→Si interaction in the silocanyl groups of oligosilanylsilocane **5** appears to be much weaker than in the silatranyl group of the oligosilanylsilatranes.

**Scheme 4:**
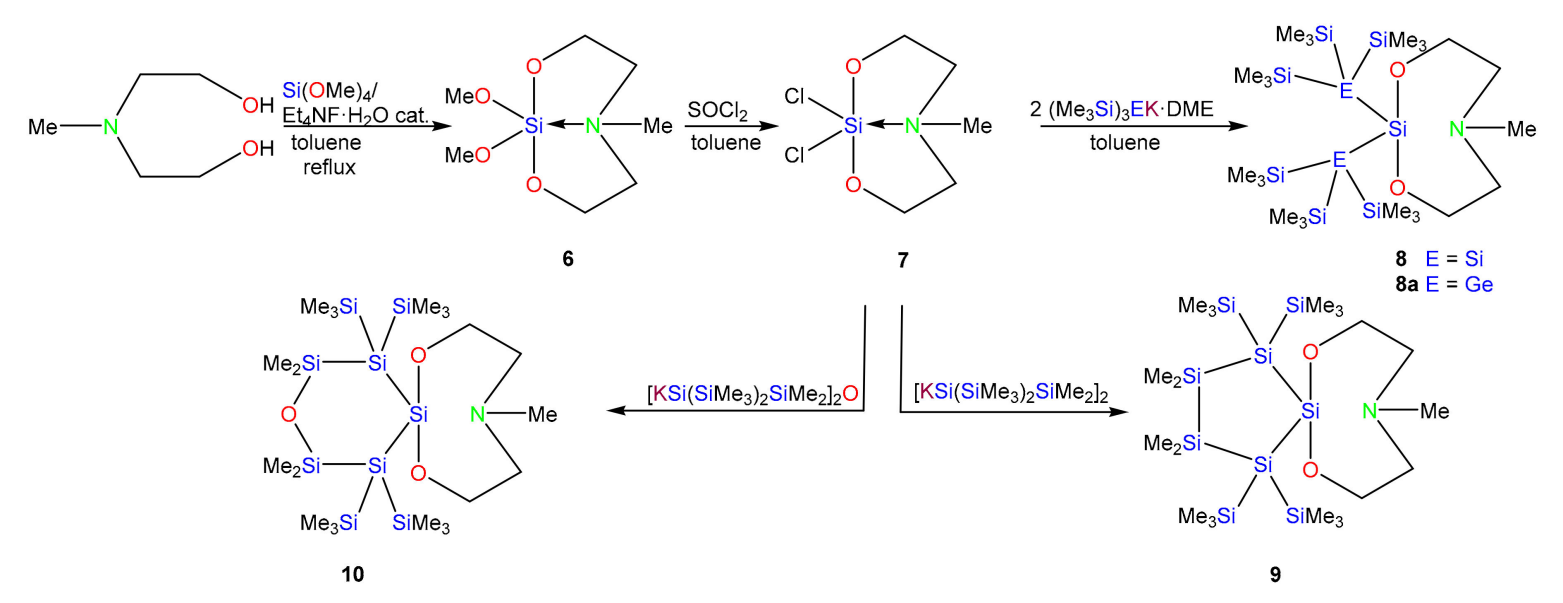
Preparation of dichlorosilocane **7** and subsequent reactions with two silanide or germanide equivalents to the oligosilanylated silocane compounds **8, 9,** and **10** as well as the digermylated compound **8a**.

Only two valences of silicon are required to form a silocane unit. For the cases of oligosilanylsilocanes **3** and **5** one of the remaining valences is occupied by the oligosilanyl moiety and the other one by a methyl group. If we wish to attach two oligosilanyl units to the silocane, the structure of chlorosilocane **2** needs to be modified in a way that the methyl group attached to the silocane silicon is formally replaced by another leaving group. These considerations suggest the synthesis of dichlorosilocane **7**. Transesterification of alkoxysilanes and alkyl alcohols in the presence of a base has been reported previously [27], thus Si(OMe)_4_ was reacted with N-methyldiethanolamine with tetraethylammonium fluoride hydrate as base to yield dimethoxysilocane **6** (Scheme 4). Due to the oily nature and sensitivity to trace amounts of moisture, the conversion of **6** to dichlorosilocane Cl_2_Si(OCH_2_CH_2_)_2_NMe (**7**) by reaction with thionyl chloride was carried out without further purification [28].

Bis(oligosilanyl)silocane **8** was then obtained by reaction of two equivalents of tris(trimethylsilyl)silyl potassium with **7** (Scheme 4). Although the (Me_3_Si)_3_Si group is relatively bulky and we have previously found that double geminal tris(trimethylsilyl)silylation can be a challenge, NMR spectroscopic analysis of the reaction mixture indicated clean formation of **8** in satisfying yields. It is interesting to note that attempted monosilylation of **7**, by reaction with one equivalent of tris(trimethylsilyl)silyl potassium, was not possible. Spectroscopic analysis showed only formation of compound **8** and residual **7**. We assume that the monosilylated product is much more soluble compared to **7**, and thus reacts preferentially with the silanide. 

**Figure 2:**
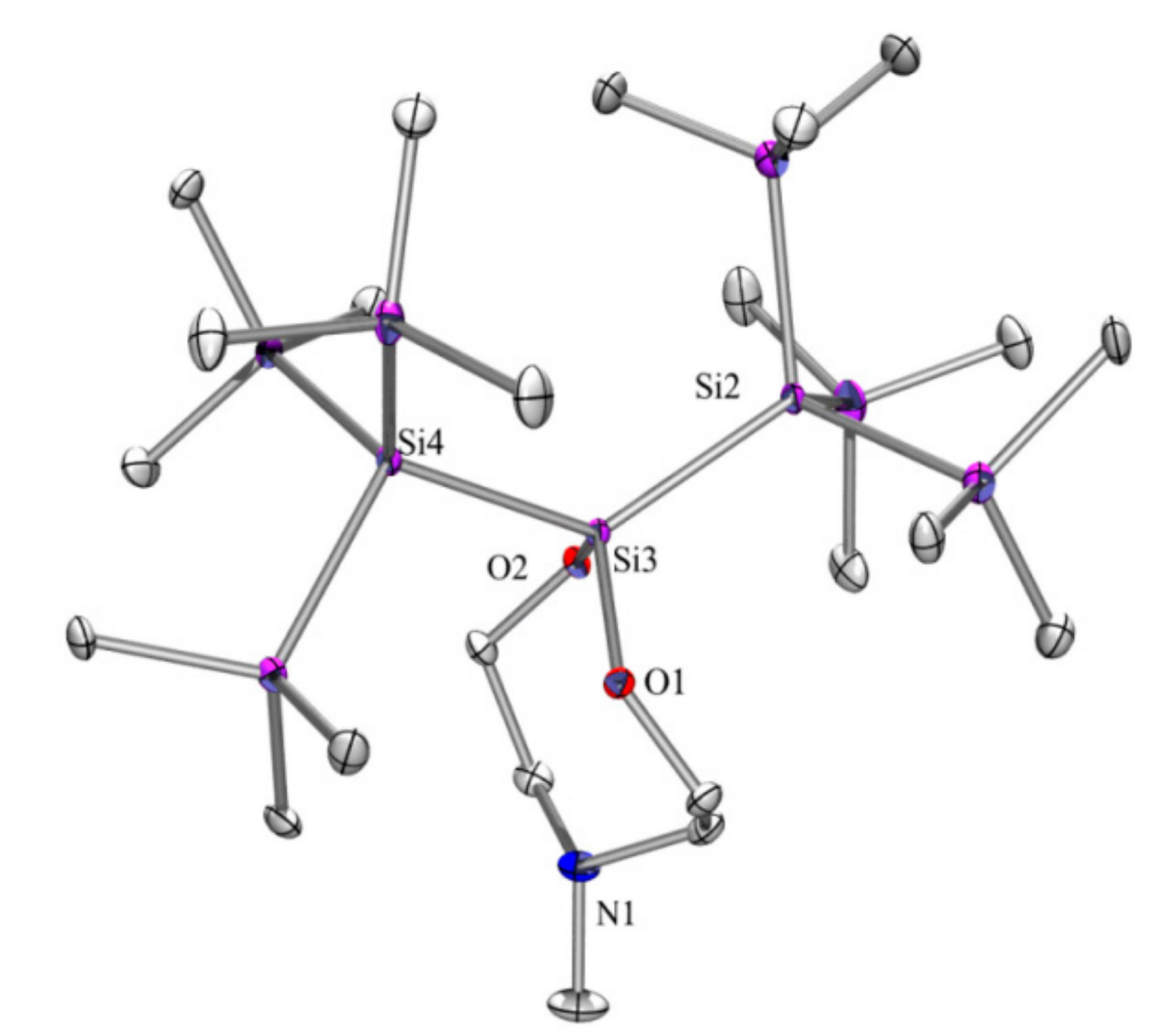
Molecular structure of **8** (thermal ellipsoid plot drawn at the 30% probability level). All hydrogen atoms are omitted for clarity (bond lengths in Å, angles in deg). Si(3)-O(1) 1.652(2), Si(3)-O(2) 1.660(2), Si(3)-Si(4) 2.3811(12), Si(3)-Si(2) 2.3881(13), Si(2)-Si(1) 2.3542(14), Si(1)-C(7) 1.887(4), O(1)-C(1) 1.433(4), N(1)-C(4) 1.459(5), C(1)-C(2) 1.510(5), O(1)-Si(3)-O(2) 110.67(12), C(4)-N(1)-C(2) 114.2(3).

Crystal structure analysis of **8** (Figure 2), which crystallizes in the orthorhombic space group *Pcc_n,_* shows that the two bulky tris(trimethylsilyl)silyl groups change the conformational properties of the silocane unit as both of the groups avoid an equatorial orientation. With N-Si-Si angles of 111.96(6) and 121.16(6) deg, both groups are located in pseudo-axial positions. The Si-N distance of 3.625 Å is considerably longer than in **3** and even longer than the Si-N distance of a protonated oligosilanylsilatrane (3.389 Å) [3] and the N-methyl group is almost aligned with the N-Si axis (Me-N-Si angle = 169.8(2) deg). This shows that no or only a very weak interaction exists between the silicon and nitrogen atoms in oligosilocanylsilane **8**. With 110.67 deg the O-Si-O angle in **8** is somewhat smaller than in **5** (113.50 degree) and much smaller than the 121.69 deg in chlorosilocane **2**. The long Si-N distance and the small O-Si-O angle in oligosilocanylsilane **8** are related to the presence of the two bulky hypersilyl groups around the silocanyl group. The Si-SiO_2_ bonds of 2.388(1) and 2.381(1) Å are longer than the analogous distance in **5** (2.3564(9) Å) or an average Si-Si bond of 2.3721 Å but are almost identical with the respective Si-Si bonds in [(Me_3_Si)_3_Si]_2_SiMe_2_ [29].

In contrast to other silocane compounds, which are sensitive to hydrolysis, bis(oligosilanyl)silocane **8** is stable in air. We attribute its enhanced stability to a shielding effect of the two bulky (Me_3_Si)_3_Si groups attached to the silocanyl silicon atom. 

In a similar way to the preparation of compound **8**, the analogous compound **8a** bearing two tris(trimethylsilyl)germyl groups was also synthesized by reaction of two equivalents of tris(trimethylsilyl)germyl potassium [30] with dichlorosilocane **7** (Scheme 4).

In contrast to the clean formation of bis(oligosilanyl)silocane **8**, NMR spectroscopic analysis of the reaction mixture indicated the presence of a substantial amount of tetrakis(trimethylsilyl)germane in addition to the formed bis[tris(trimethylsilyl)germyl]silocane **8a**. 

**Figure 3:**
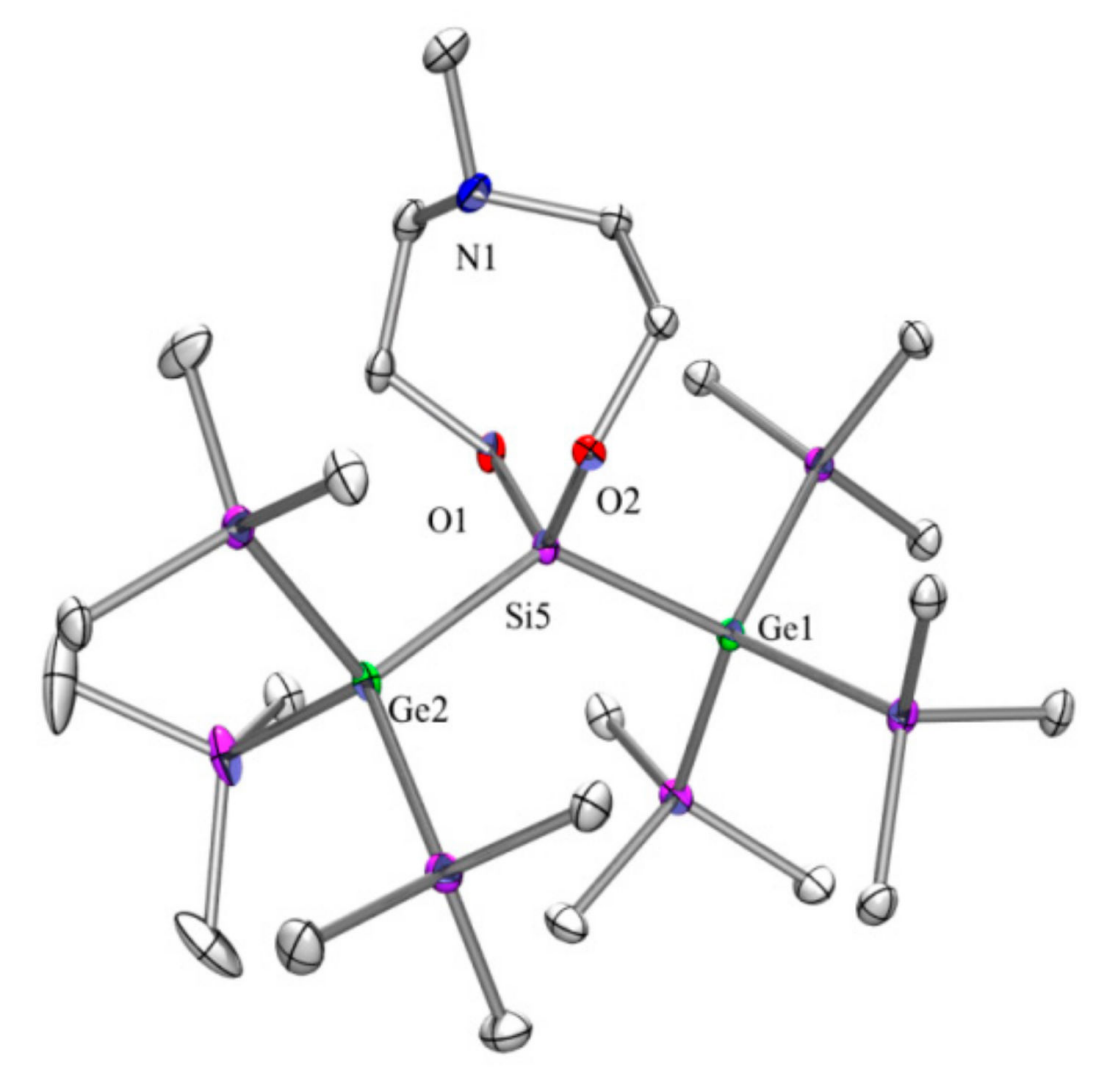
Molecular structure of **8a** (thermal ellipsoid plot drawn at the 30% probability level). All hydrogen atoms are omitted for clarity (bond lengths in Å, angles in deg). Ge(1)-Si(2) 2.3863(6), Ge(1)-Si(5) 2.4105(6), Ge(2)-Si(5) 2.4177(5), N(1)-C(2) 1.461(2), O(1)-C(1) 1.431(2), O(1)-Si(5) 1.6544(12), O(2)-Si(5) 1.6501(12), Si(2)-C(6) 1.876(2), C(1)-C(2) 1.515(3), Si(2)-Ge(1)-Si(3) 110.717(19), C(2)-N(1)-C(5) 113.03(15), O(2)-Si(5)-O(1) 112.30(6), Ge(1)-Si(5)-Ge(2) 126.99(2).

The crystal structure of **8a** (Figure 3), which crystallizes in the triclinic space group P-1 is somewhat different from that of **8**. While the Ge-Si-Ge angle of 126.99(2) is fairly close to that of **8** (126.77(5) deg), the N-Si-Ge angles of 107.77(3) and 125.23(3) deg indicate a different silocane ring conformation. This is confirmed by an Si-N distance of 3.501(1) Å, which is substantially shorter than the 3.625(3) Å found for oligosilocanylsilane **8**. The O-Si-O angle of 112.30(6) deg in oligosilocanylsilylgermane **8a** is slightly wider than O-Si-O angle in oligosilocanylsilane **8** with 110.7(1) deg. Nevertheless, the long Si-N distance and narrow O-Si-O angle suggest that similar as for oligosilocanylsilane **8** also no Si-N interaction exists in oligosilocanylsilylgermane **8a**. As expected, the exocyclic Si-Ge distances of **8a** (2.4105(5) and 2.4177(6) Å) are slightly elongated compared to the Ge-SiMe_3_ distances, which span a range between 2.386 and 2.416 Å.

Accordingly, oligosilocanylsilylgermane **8a** is also stable to ambient conditions which is related again to the shielding effect of two the bulky (Me_3_Si)_3_Ge- groups around the silicon atom of the silocanyl unit.

Similar to the syntheses of **8** and **8a**, the novel spirocyclic compounds **9** and **10** were synthesized by the reactions of **7** with 1,4- and 1,5-dianionic oligosilanes K(Me_3_Si)_2_(SiMe_2_)_2_Si(SiMe_3_)_2_K [31] and K(Me_3_Si)_2_SiMe_2_OSiMe_2_Si(SiMe_3_)_2_K [6] (Scheme 4), respectively. 

**Figure 4:**
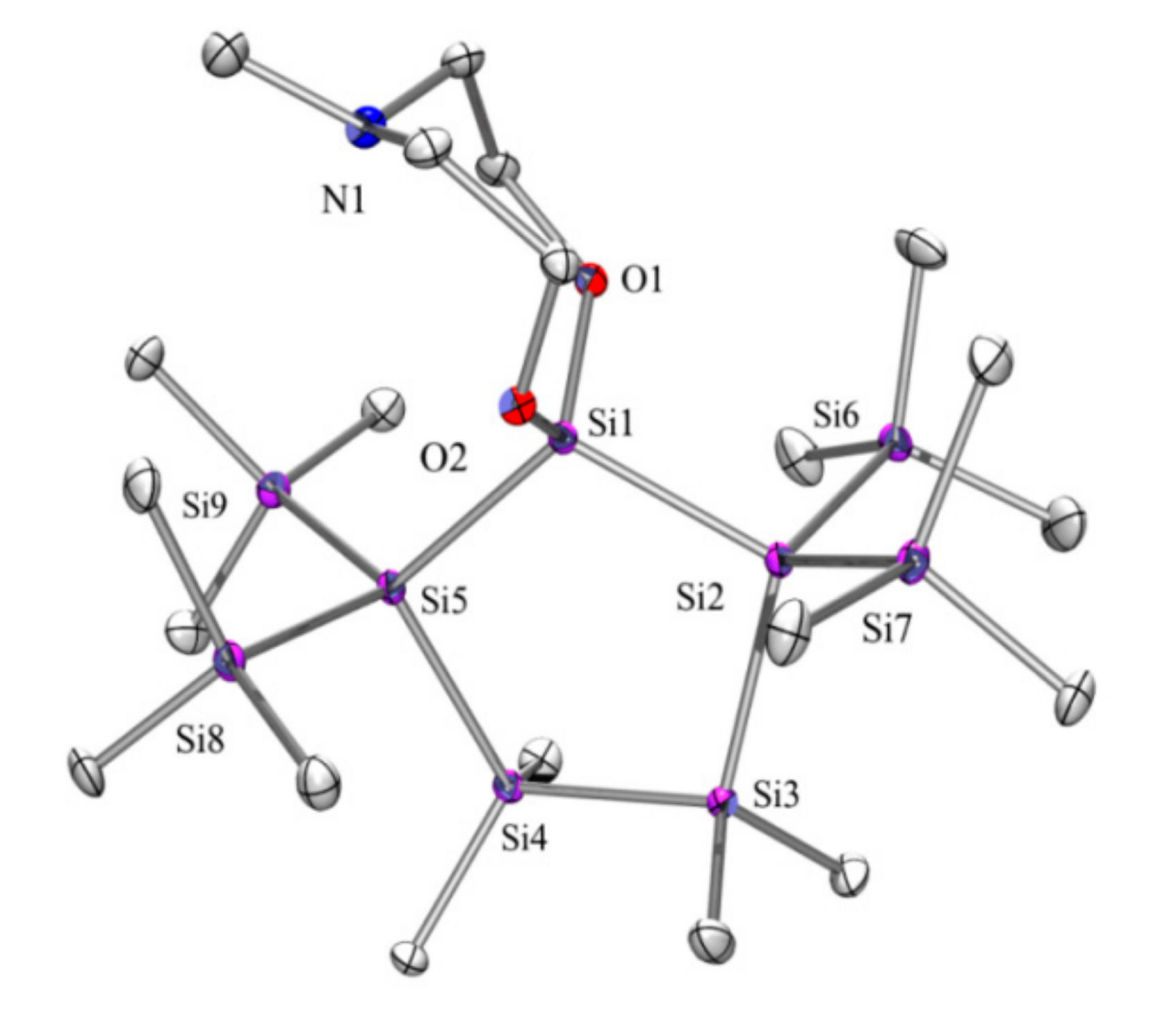
Molecular structure of **9** (thermal ellipsoid plot drawn at the 30% probability level). All hydrogen atoms are omitted for clarity (bond lengths in Å, angles in deg). Si(1)-O(1) 1.656(2), Si(1)-O(2) 1.659(2), Si(1)-Si(2) 2.3553(12), Si(3)-C(6) 1.894(3), N(1)-C(4) 1.461(4), O(1)-C(1) 1.426(4), O(2)-C(3) 1.422(3), C(1)-C(2) 1.508(4), O(1)-Si(1)-O(2) 109.92(11), Si(2)-Si(1)-Si(5) 109.59(5), C(21)-N(1)-C(4) 111.6(3), C(4)-N(1)-C(2) 114.8(3).

**Figure 5:**
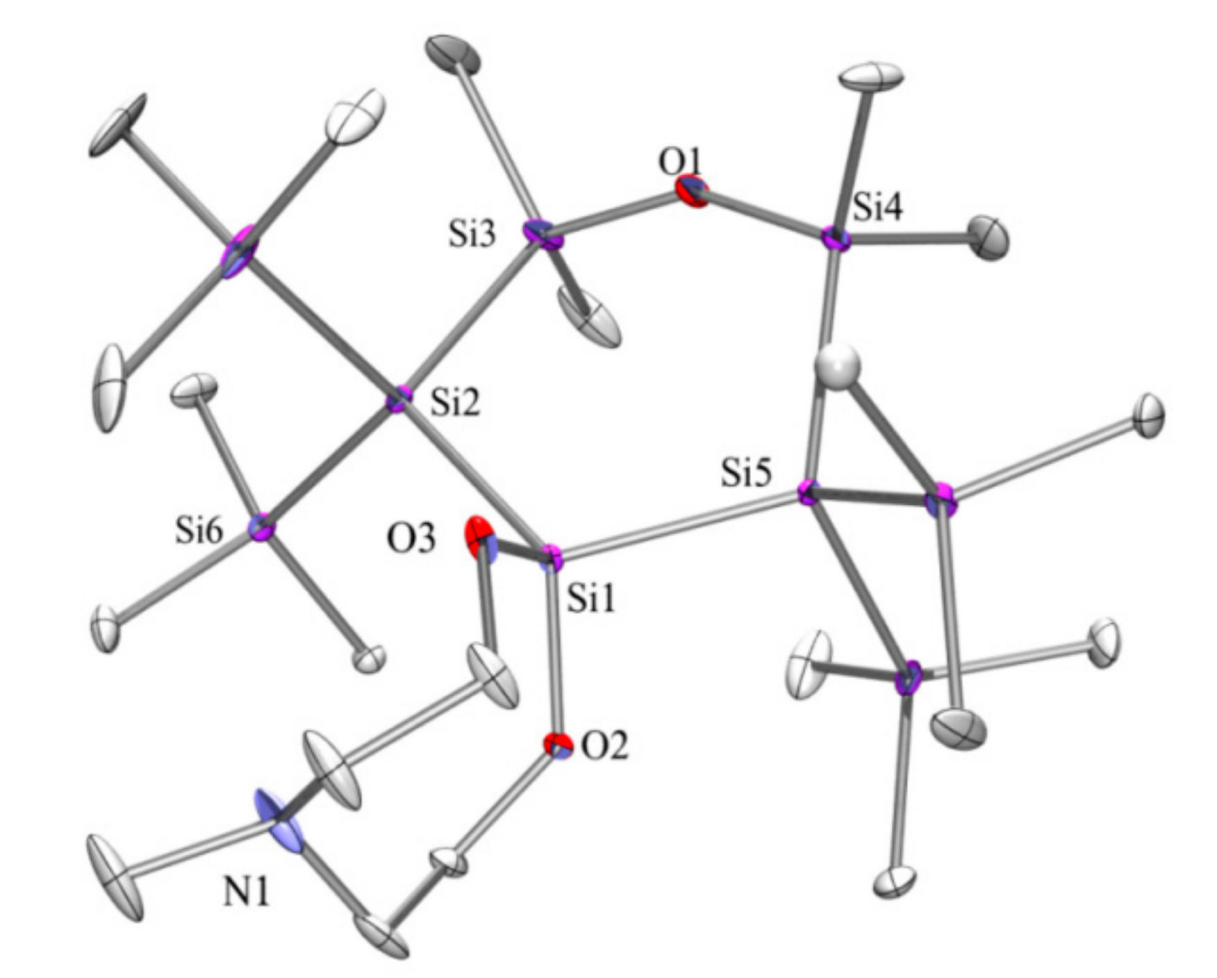
Molecular structure of **10** (thermal ellipsoid plot drawn at the 30% probability level). All hydrogen atoms are omitted for clarity (bond lengths in Å, angles in deg). Si(1)-O(2) 1.644(2), Si(1)-O(3) 1.654(2), Si(1)-Si(2) 2.3627(13), Si(2)-Si(6) 2.3499(13), Si(3)-O(1) 1.630(3), Si(3)-C(1) 1.864(6), Si(4)-O(1) 1.621(3), N(1)-C(21) 1.453(8), N(1)-C(17) 1.456(6), O(2)-C(20) 1.417(4), O(2)-Si(1)-O(3) 110.63(14), C(21)-N(1)-C(17) 114.1(5), Si(4)-O(1)-Si(3) 150.49(18).

The spirocyclic structures of **9** (Figure 4) and **10** (Figure 5) feature due to the cyclic nature of the oligosilanyl part smaller Si-SiO_2-_Si angles (109.59(4) and 115.03(5) deg, respectively) than **8** and **8a**. Somewhat surprising the O-Si-O angles of **9** and **10** (109.9(1) and 110.6(1) deg) are also smaller than those of **8** and **8a** (110.7(1) and 112.30(6) deg). Both cyclosilane units feature envelope conformations with one of the SiMe_2_ units as flaps. Although the cyclosilane part of **10** is formally a six-membered ring, the wide Si-O-Si angle of 150.5(2) deg causes the Si-O-Si unit to function as one edge of the ring. 

With the availability of compounds **8**, **9,** and **10** we were interested whether these could be converted to oligosilanides or oligosilane diides. The clean conversion of the singly oligosilanylated compound **3** to silanide **4 [6]** suggested that the same could be done with **8**, **9,** and **10**. 

**Scheme 5:**
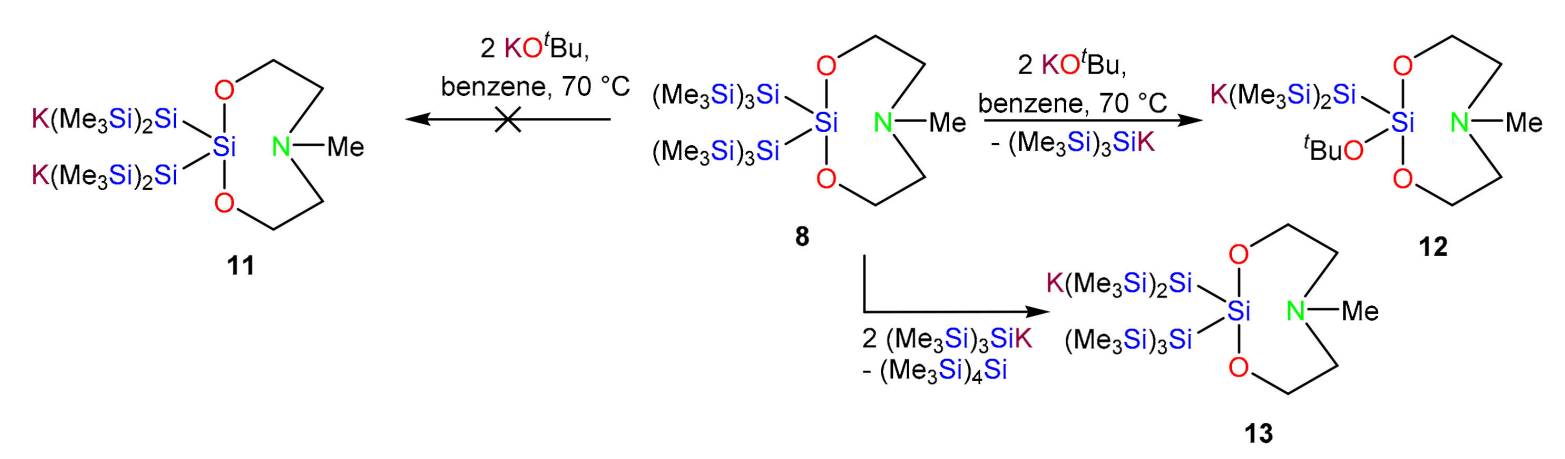
Attempted formation of silocanyloligosilane diide **11**, by reaction of **8** with two equiv KO*^t^*Bu. Instead, *tert-*butoxysilocanyloligosilanide **12** was obtained. Reaction of **8** with tris(trimethylsilyl)silyl potassium led to the formation of monosilanide **13**.

However, reaction of **8** with two equivalents of KO*^t^*Bu in benzene at 70 °C did not give the expected oligosilane diide **11** but rather a mixture of mono-anionic compound **12** and tris(trimethylsilyl)silyl potassium (Scheme 5). It seems that the silocane silicon atom of **8** is not only fairly electrophilic, it is also sterically accessible with the two long Si-Si bonds and the weak interaction with the nitrogen atom. Therefore, attack of the alkoxide does not occur at one of the six trimethylsilyl groups but rather at the silocane silicon atom, generating a Si-O*^t^*Bu bond with the tris(trimethylsilyl)silanide as leaving group. The attack of the second *tert-*butoxide eventually occurs at one of the three remaining trimethylsilyl residues to form silanides **12** (Scheme 5). In an attempt to change regioselectivity of the alkoxide attack, the reaction was repeated at ambient temperature and in the presence of two equivalents of 18-crown-6. The outcome was, however, the same. As we frequently observe silyl-metal exchange behavior between oligosilanides and oligosilanes, we decided to react compound **8** with two equivalents of tris(trimethylsilyl)silyl potassium in DME. Indeed, the reaction proceeded in a way that the oligosilanyl-silocane bonds remain intact but it did not proceed to the desired oligosilane diide **11**, instead stopping at the mono-silanide stage **13** (Scheme 5).

**Figure 6:**
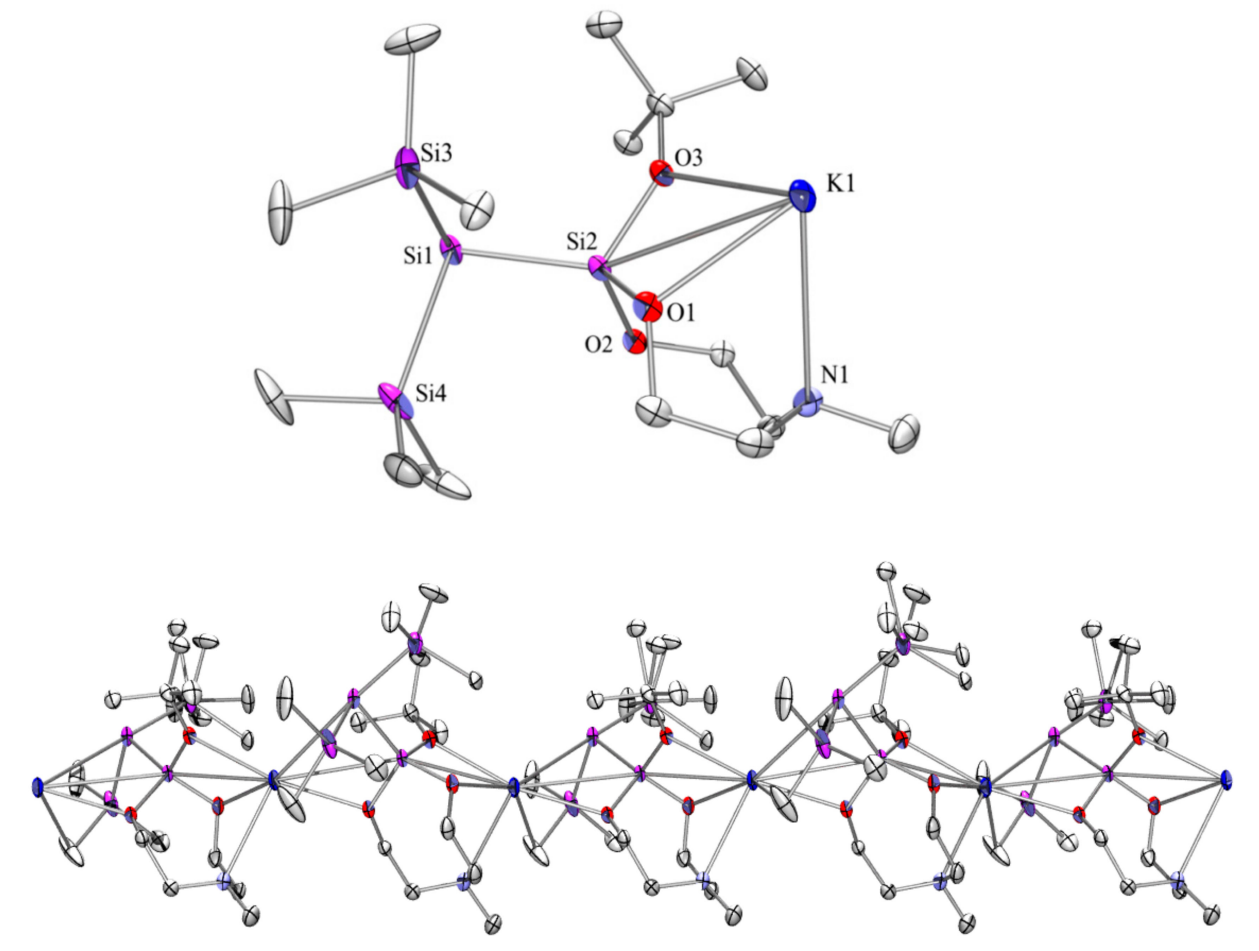
Molecular structure of **12** (thermal ellipsoid plot drawn at the 30% probability level) above and packing plot below. All hydrogen atoms are omitted for clarity (bond lengths in Å, angles in deg.). K(1)-O(1) 2.692(2), K(1)-O(3) 2.699(2), K(1)-O(2)#1 2.727(2), K(1)-N(1) 2.990(3), K(1)-Si(1)#1 3.4702(11), K(1)-Si(2) 3.4942(11), 1.437(4), O(2)-Si(2) 1.683(2), O(3)-Si(2) 1.664(2), Si(1)-Si(2) 2.3052(12), Si(1)-Si(4) 2.3437(13), Si(1)-Si(3) 2.3544(12), Si(2)-Si(1)-Si(4) 102.27(5), Si(2)-Si(1)-Si(3) 99.17(4), Si(4)-Si(1)-Si(3) 100.73(5), O(3)-Si(2)-O(1) 95.56(10), O(3)-Si(2)-O(2) 106.19(10), O(1)-Si(2)-O(2) 104.11(11).

The structure of **12** (Figure 6) is similar to that of a silatranylsilanide as is quite evident from its NMR spectroscopic properties (vide infra). The solid state structure of **12** consists of a coordination polymeric chain where the potassium ion bridges two silanide units coordinating to the nitrogen atom, one CH_2_O unit, and the *tert*-butoxy oxygen atom of one entity and to the other CH_2_O oxygen atom and the silanides Si of the neighboring atom. Distances of ca 2.7 Å to oxygen, 3.0 Å to nitrogen, and 3.4 Å to silicon are within the typical ranges observed. The silanide’s silicon atom is fairly pyramidalized (sum of Si-Si-Si angles is 302.17(5) deg).

**Scheme 6:**
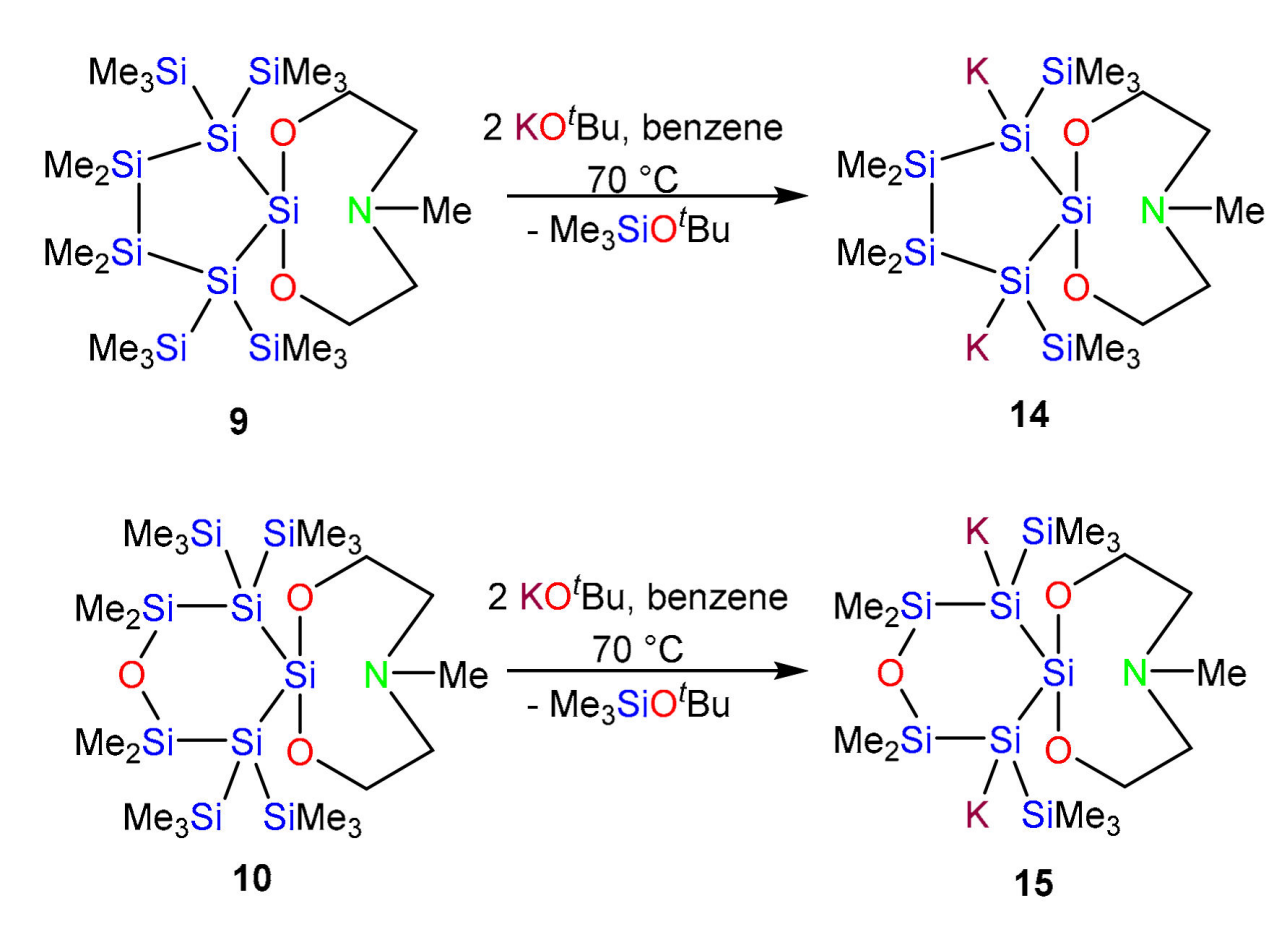
Conversion of spirocyclic oligosilanylsilocanes **9** and **10** to the respective oligosilane diides **14** and **15**.

When we tried to prepare oligosilanyldiides from **9** and **10** by reaction with two equiv KO*^t^*Bu in benzene at 70 °C we succeeded in the isolation of the desired compounds **14** and **15** (Scheme 6). Attempts to conduct the reaction in THF as solvent led to cleavage of the (Me_3_Si)_2_Si-SiO_2_ bond and the addition of 18-crown-6 to benzene, leading to messy reactions. Pure compounds **14** and **15** crystallized directly from the reaction mixtures, after 1 d at 70 °C in benzene.

### 2.2. NMR Spectroscopy 

**Scheme 7:**
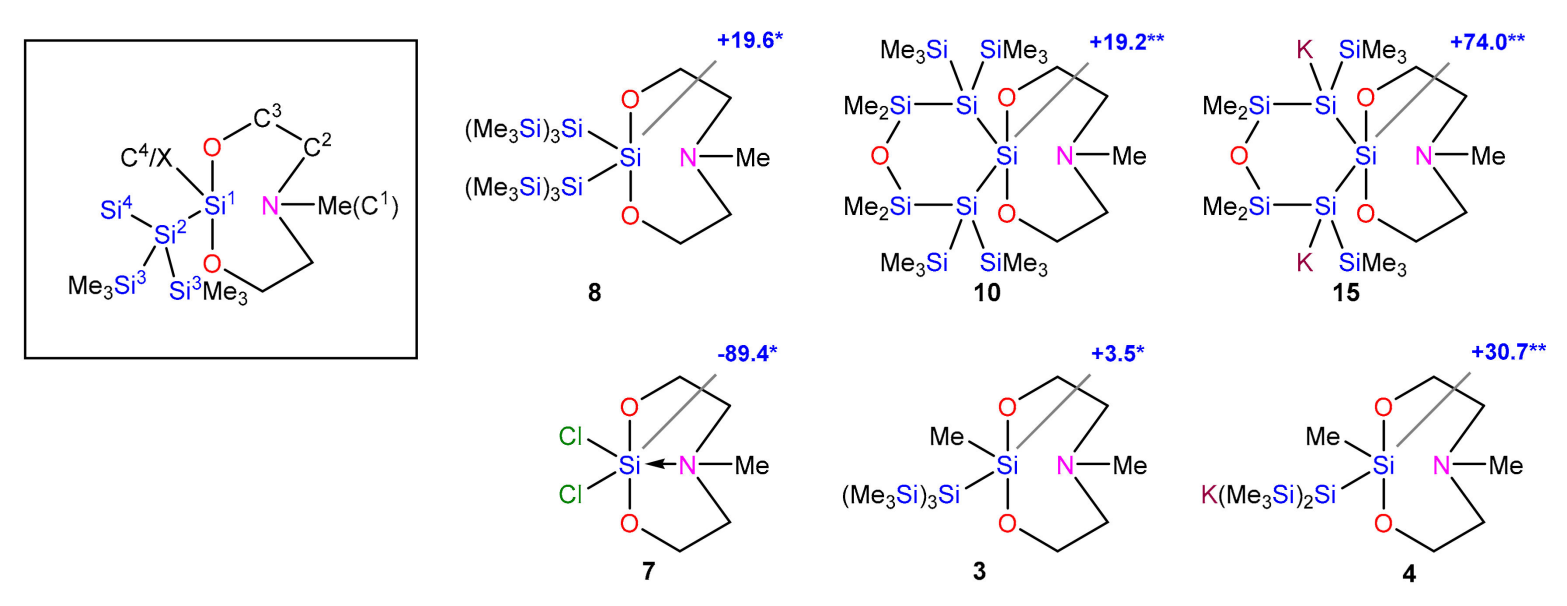
^29^Si-NMR chemical shifts of quaternary SiO_2_ fragments in ppm in CDCl_3_ * or C_6_D_6_ **. Numbering scheme for Table 1 is given in the boxed structure.

All compounds of this study were characterized by multinuclear NMR spectroscopy. With respect to structural properties, arguably the most important nucleus is ^29^Si but the ^13^C data are also interesting (Table 1, Scheme 7). The chemical shifts of the nitrogen-attached methylene units (C^2^) are surprisingly sensitive to the silocane substituents. Although the values for **2**, **6**, and **7** are around δ*_C__2_* = 54 ppm, silyl substitution causes an approximate 5 ppm shift to values around δ*_C__2_* = 59 ppm (Table 1). A similar trend is also visible for the oxygen-attached methylene units (C^3^), which are found close to δ*_C__3_* = 60 ppm for **2**, **6**, and **7** but again experience a downfield shift to values around δ*_C__3_* = 65 ppm for the silyl substituted examples. Somewhat unexpected silanide formation, such as is the case for **4**, **12**, **14**, and **15,** does not really affect the chemical shifts of C^2^ and C^3^. The signal for the methyl group at the silocane Si at compounds **2**–**5**, occurs close to 4 ppm for the neutral compounds **2**, **3**,and **5** but shifts slightly to 6.4 ppm for the silanide **4**. 

The ^29^Si-NMR chemical shift of the quaternary SiO_2_ fragment is most strongly influenced by its chemical environment exhibiting values ranging from δ*_Si_* = −91.7 ppm for the dimethoxy compound **6** to δ*_Si_* = +91.5 ppm for **14** (Table 1, Scheme 1). The situation is somewhat alike to that of the analogous silatranes. The chemical shift of chlorosilatrane (−85.9 ppm) is similar to that of **6** and **7**. The signals of **3** (+3.5 ppm) and **8** (+19.5 ppm) reflect the downfield shift associated with the change of an alkoxy substituent for either a methyl or oligosilanyl group. One unusual feature of the silatranyloligosilanide NMR spectroscopy was the unusually strong downfield shift of the silatranyl Si signal upon silanide formation [3,5]. Starting from a resonance for the silatranyl silicon atom of tris(trimethylsilyl)silylsilatrane of δ*_Si_* = −52.8 ppm, the respective silanides obtained by abstraction of a trimethylsilyl group resonated at −11.8 ppm, yielding a ∆δ*_Si_* of +40.8 ppm. Comparison with the conversion of (MeO)_3_*Si*Si(SiMe_3_)_3_ (δ*_Si_* = −32.2 ppm) to (MeO)_3_*Si*Si(SiMe_3_)_2_K (δ*_Si_* = +0.9 ppm) reveals a downfield shift of ∆δ of +33.1 ppm. Therefore, the change of Si^1^ from **3** (δ*_Si_*_1_ = +3.7 ppm) to **4** (δ*_Si_*_1_ = +35.9 ppm) corresponding to a ∆δ*_Si_*_1_ of +32.4 ppm is completely within the range of expectation. More interesting, however, is that a double α-anionic substituent causes a really substantial shift of ca. 55 ppm as evidenced by the transformations of **9** to **14** (∆δ*_Si_*_1_ = +58.4 ppm) and **10** to **15** (∆δ*_Si_*_1_ = +54.8 ppm). A ^29^Si chemical shift of δ*_Si_*_1_ = 91.5 ppm, as observed for the silocane Si^1^ by **14,** is typically associated with a high degree of electrophilicity [32].

Compound **12** is special as it has the substitution pattern of (MeO)_3_*Si*Si(SiMe_3_)_2_K or silatranyloligosilanide (RO)_3_*Si*Si(SiMe_3_)_2_K. The silocanyl Si^1^ resonance of δ*_Si_*_1_ = −13.9 ppm is very close to the respective silatrane [3,5] and even the strong upfield shift of the silanide signal at δ*_Si_*_2_ = −210.2 ppm (Table 1) is something we also observed for the silatranyloligosilanide cases [3,5]. 

### 2.3. Electrochemical Studies

#### 2.3.1. Oxidation

**Figure 7:**
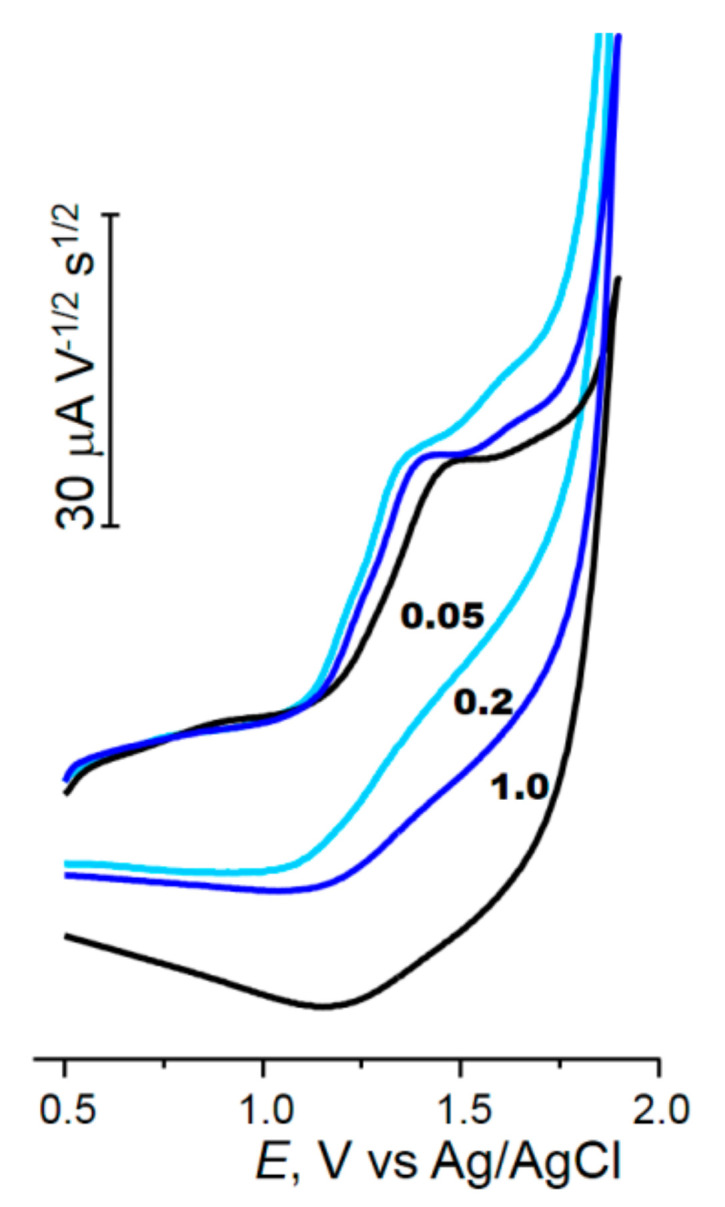
Scan rate-normalized voltammograms of oxidation of **9** at a glassy carbon (GC) electrode in AN/0.1 M Bu_4_NPF_6_. Numbers: scan rate *v* in V s^−1^.

**Figure 8:**
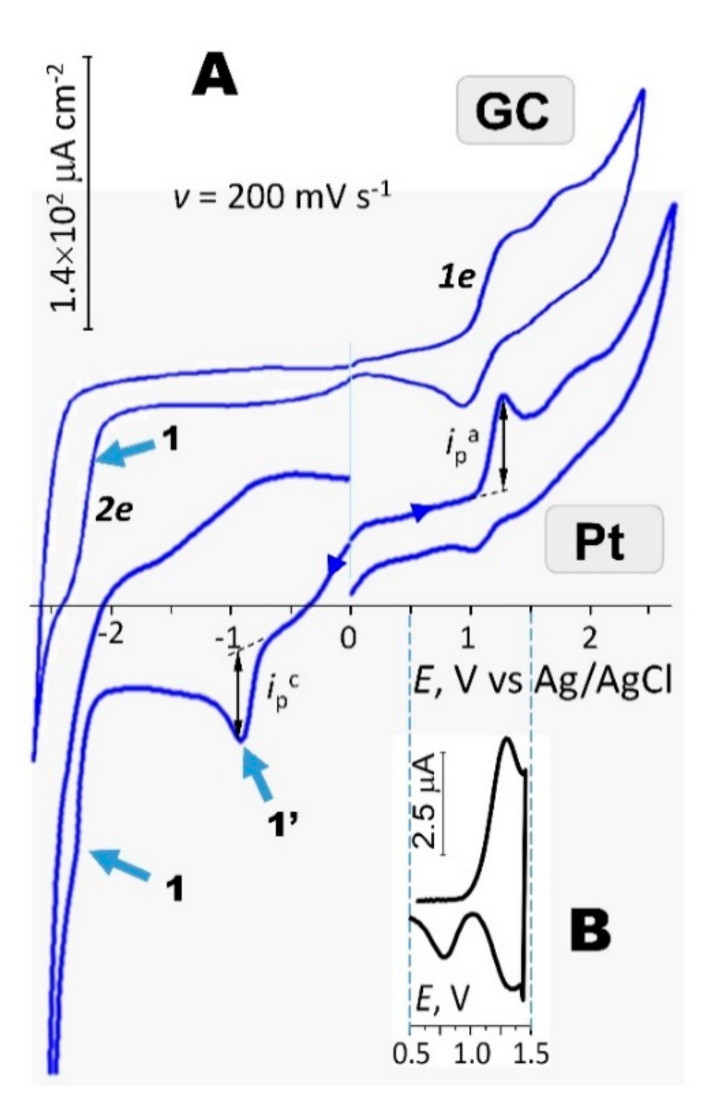
Cyclic (**A**) and square-wave (**B**) voltammetry of **7** in AN/0.1 M Bu_4_NPF_6_. T = 20 °C. Anodic and cathodic scans are traced independently.

The N→Si dative interaction that can take place in silocanes [33] directly affects the donor properties of the p_z_-type lone pair of the nitrogen atom attenuating its availability for ionization. We therefore considered electrooxidation of silocanes **5**, **7**, **8**, and **9** since their *E*_p_^ox^ supposedly reflects the occurrence and degree of this intramolecular coordination. All these silocanes showed distinct diffusion-controlled (*i*_p_/C*v*^1/2^ = const), one-electron oxidation steps within 1.0–1.4 V vs. Ag/AgCl (Figure 7 and Figure 8), followed by a smaller broad second oxidation peak at about 500 mV higher potentials. Although no reverse peaks were seen on the voltammograms of **5**, **7**, and **8** up to *v* = 5 V s^−1^, square-wave cyclic voltammetry (SWCV) attested electron transfer (ET) during the oxidation of these silocanes to be reversible. The voltammograms of **9** already revealed electrochemical reversibility at *v* > 0.5–1 V/s (*i*_p_^c^/*i*_p_^a^ > 0) (Figure 7), however further increase of the scan rate did not cause an increase in *i*_p_^c^ since ET kinetics starts to limit the process.

The oxidation of dichlorosilocane **7** occurs at E_p_ = 1.32 V vs. Ag/AgCl under diffusion control (lg(*i*_p_)/lg(*v*) = 0.48, n = 1). The oxidation peak (Figure 8) is somewhat broad (peak half-width ∆E_p-p/2_ = 104 mV) and its cathodic counterpart on the reverse scan appears negatively shifted by 250 mV (E_p_^c^ = −1.08 V). This feature suggests the oxidation of **7** to be quasi-reversible [34], with slow ET kinetics leading to a cation radical and a large structural reorganization energy accompanying the process. Broadened peaks (∆E_p-p/2_ ≅ 80–90 mV) and an increased slope ∆E_p_/∆lg(*v*) = 63 mV for **8** and **9** (35 mV for **7**) also attest to slow kinetics of ET. 

The *E*_p_s of the silocanes (Table 2) have intermediate positions between the *E*_p_ of trialkyl amines, e.g., Et_3_N (0.78 V vs. SCE) [35] and silatranes [4,36,37,38,39], which at first sight might reflect a lesser extent of involvement of their nitrogen p_z_ electrons into 3c-4e hyperbonding than in silatranes. However, a closer look reveals that the situation is more complex. 

**Figure 9:**
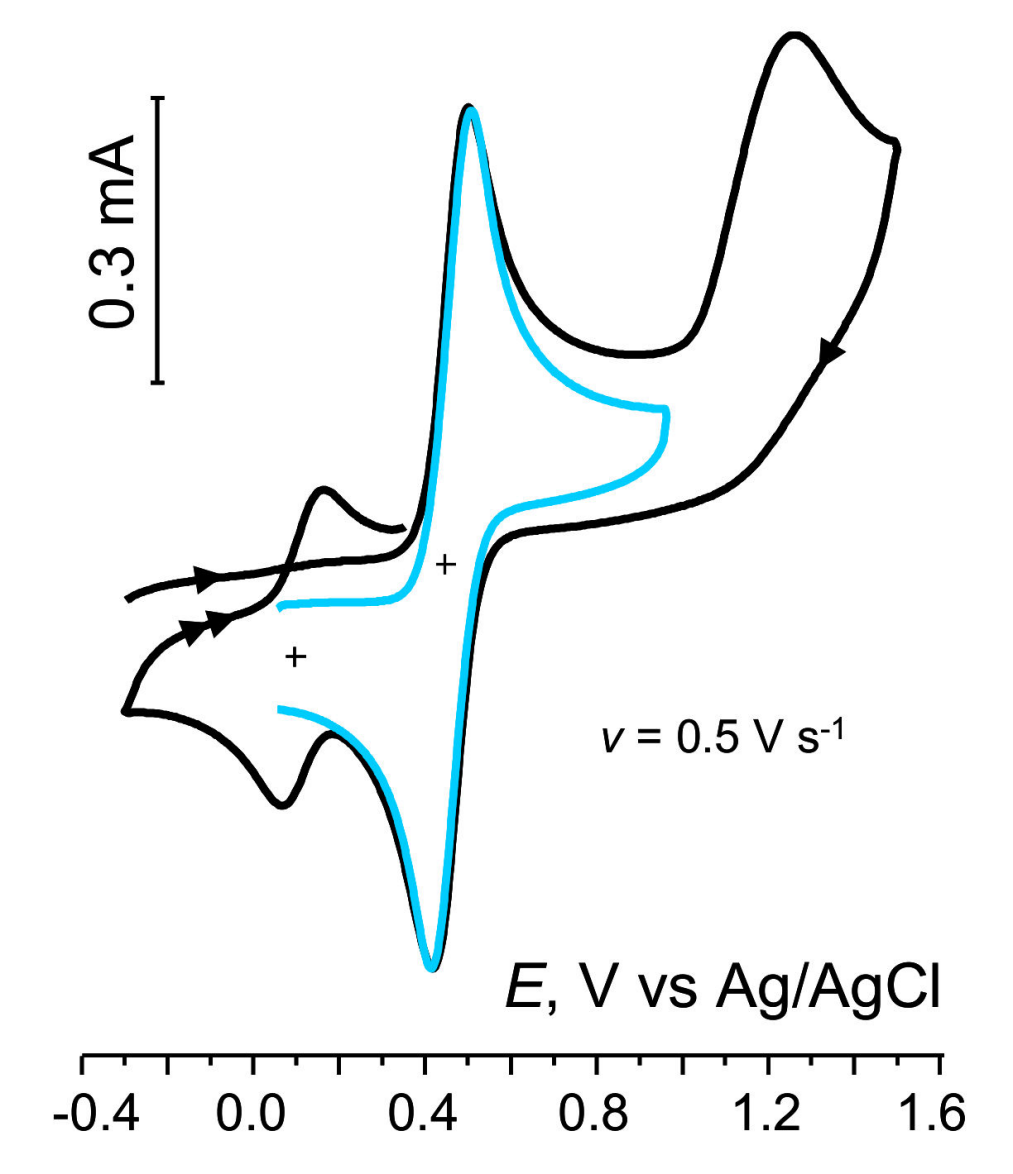
Oxidation of ferrocene alone (blue) and in the presence of **7** (black) at a GC electrode in AN/0.1 M Bu_4_NPF_6_. Cross marks show the *E*_0_s of the two redox systems.

When the vertex potential in SWCV is set between *E*_p_^1^ and *E*_p_^2^, one can see two reductions associated to the first electron withdrawal from **7** (Figure 8B), the new one (*E*_p_^c^ = 0.75 V) corresponding to the redox system **7**^•+^/**7** with an adiabatically rearranged CR without N→Si coordination while that at 1.25 V is related to the CR in its initial configuration. The situation therefore resembles the oxidation of silyl-substituted silatranes when both coordinated (*endo*) and noncoordinated (*exo*) forms of their CRs have been detected by SWCV [4]. The formation of an *exo*-amine form of **7**, which appears after the removal of one electron from the 3c-4e system (HOMO of *endo*-form) destroying its bonding character, can be demonstrated by trapping this *exo*-form via complexation with ferrocene [37] leading to a new redox system at *E*_0_ = 0.1 V, 300 mV easier to oxidize than Fc itself (Figure 9).

In spite of two strong acceptor substituents at Si in **7**, which could expectedly increase its *E*_p_, as in case of silatranes (for chlorosilatrane, *E*_p_ = 2.05 V vs. SCE) [37], its oxidation occurs at very moderate anodic potentials and its *E*_p_ is comparable to the *E*_p_s of **8** and **9** (Table 2), Taking no account of a specific interaction N→Si and using the E_p_-Σσ* correlation for trialkylamines [40] with Taft’s σ*-constants [41] of the substituents at N, one might expect the *E*_p_ of **7** to be about 1.0-1.1 V. The fact that its *E*_p_ is just slightly more positive than those of tertiary amines [42], but less positive than *E*_p_s of silatranes [36,37], can be interpreted as corroborating the occurrence of 3c-4e hyperbonding in **7**, yet with a much weaker degree of involvement of the n(p_z_) lone pair of the N atom in a dative interaction with Si compared to silatranes.

For steric reasons (repulsion of N-Me and Si-SiMe_3_ groups), the N lone pairs of **8** and **9** (Figure 2 and 4) are practically orthogonal to the Si-N axes so they cannot participate in N→Si dative interaction and form complex 3c-4e internal systems. The HOMO in these cases is mostly formed with n(p_z_) electrons of N. Consequently, in oxidative ET, **8** and **9** behave as trialkylamines [35,42] since the ionization potential of CH_3_N(CH_2_-)_2_ unit is lower than that of polysilane chains (for oxidation of polysilanes see [43,44,45,46]). Their two step oxidation—first electron withdrawal from silocane N atom, while second being the oxidation of the oligosilanyl substituent—is thus akin to the oxidation scheme reported for Fc-substituted polysilanes [47]. 

The oxidation of bis-silocane **5** has the lowest *E*_p_ and highest ET rate (*k*_s_ = 3 × 10^−2^ cm s^−1^) in this series and, even more surprisingly, has a one-electron character (Table 2) despite the presence of two potentially electroactive silocane units. This is contrasting to two 1e steps observed for the analogous bis-silatrane [4] and is rather typical for ET from a common HOMO.

#### 2.3.2. Reduction 

Silocanes **5**, **8**, and **9** are formed solely with σ-bonds, whose lowest lying electron-deficient σ*(Si-SiO_2_) component is involved into the interaction with donor p_z_ doublet of N. Consequently, these silocanes have no vacant orbital of appropriately low energy for accommodating an extra electron, so they are not reducible within the window of conventional CV potentials (up to −3.0… −3.3 V vs. SCE). In fact, if longer wavelength UV transition at λ = 254-260 nm for **5**, **8**, and **9** corresponds to HOMO-LUMO promotion (4.76–4.88 eV), then with an *E*_p_^ox^ ≅ 1.3 V their reduction is expected to occur at E ≅ −3.5 V, slightly beyond the limit of the AN/TBA^+^ media.

**Figure 10:**
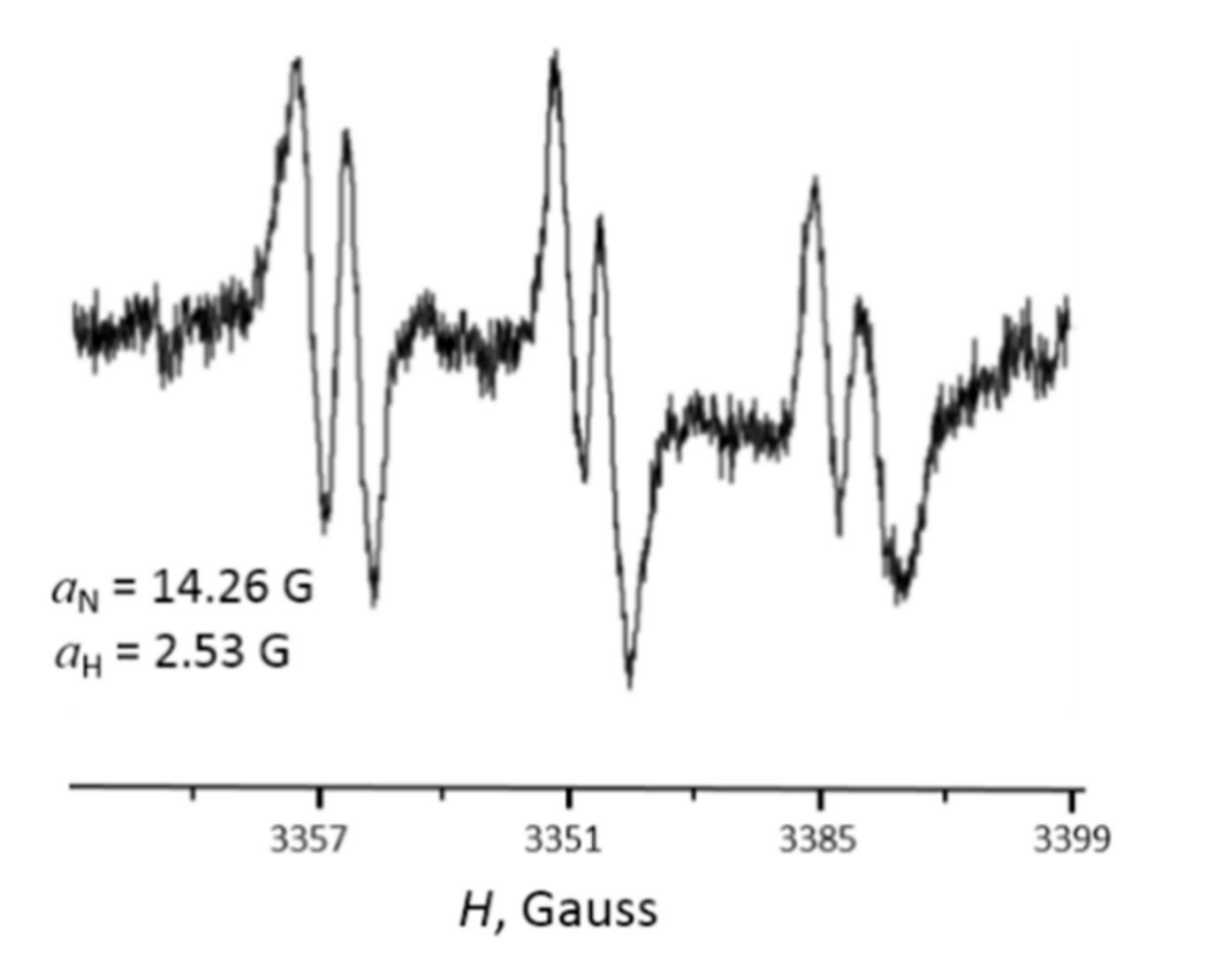
EPR spectrum of the spin adduct from the reduction of **7** at a Pt electrode (E = −0.9 V) in the presence of N-*t*-butyl-α-phenylnitrone (PBN). AN/0.1 M Bu_4_NPF_6_.

In contrast to **5**, **8**, and **9**, two σ*(Si-Cl) orbitals in dichlorosilocane **7** are suitable for accepting electrons, which renders **7** reducible. Moreover, two fashions of reduction were observed (Figure 8), depending on the electrode used. At glassy carbon (GC), having no specific affinity toward Si-Cl compounds, the reduction occurs at *E*_p_ = −2.26 V (n = 2), in the range of typical values of *E*_p_ for chlorosilanes (for Me_2_SiCl_2_, *E*_p_ = −2.2 V) [48]. At a Pt electrode, an additional reduction step (1′) is observed at substantially less negative potentials (E_p_ = −0.91 V), corresponding to a one-electron reduction of a Me(CH_2_CH_2_O)_2_NSi(Cl)-Pt(II)-Cl species formed through oxidative addition at a Pt cathode similarly to what was reported for the reduction of (EtO)_3_SiCl [49]. The formation of a Si-centered silocanyl radical at this step was confirmed by its trapping with N-*t*-butyl-α-phenylnitrone (PBN) and ex-cell EPR spectroscopy of the resulting radical adduct (Figure 10).

Interestingly, *E*_p_(**7**) at GC is more negative than that of Cl-silatrane (*E*_p_ = −2.05 V vs. SCE) [37] though the E_p_ of the latter is negatively shifted by the electron-donor atrane cage so that in the absence of this effect it is expected to be more positive. The negative shift of *E*_p_(**7**) supposedly originates from the geminal exchange between the two Si-Cl bonds and delocalization of the electron density from the σ(Si-Clax) bond, whose energy is pushed up by N→Si-Cl_ax_ 3c-4e interaction, into the equatorial σ*(Si-Cleq) orbital (site of ET); a similar effect was earlier found for N-phenyl dichlorosilocane [17]. The exact value of *E*_p_(**7**) is evidently determined by a complex interplay of fine electronic interactions related to geometry, coordination, cycle strain and external coordination interactions.

#### 2.3.3. DFT Study

The rationalization of the results of the electrochemical study was completed by DFT analysis of these systems. DFT B3LYP/aug-CC-pVQZ optimized geometries of **8** and **9** correspond well to those from X-ray diffractometry, showing both *l*_N-Si_ distance and p_z_(N) orbital orientation to be prohibitive of the formation of a 3c-4e system in these silocanes. Therefore, HOMO of **8** and **9** is formed of the n(p_z_) lone pair of N, which makes their oxidation similar to that of trialkylamines, in agreement with the CV behavior of these compounds. 

**Figure 11:**
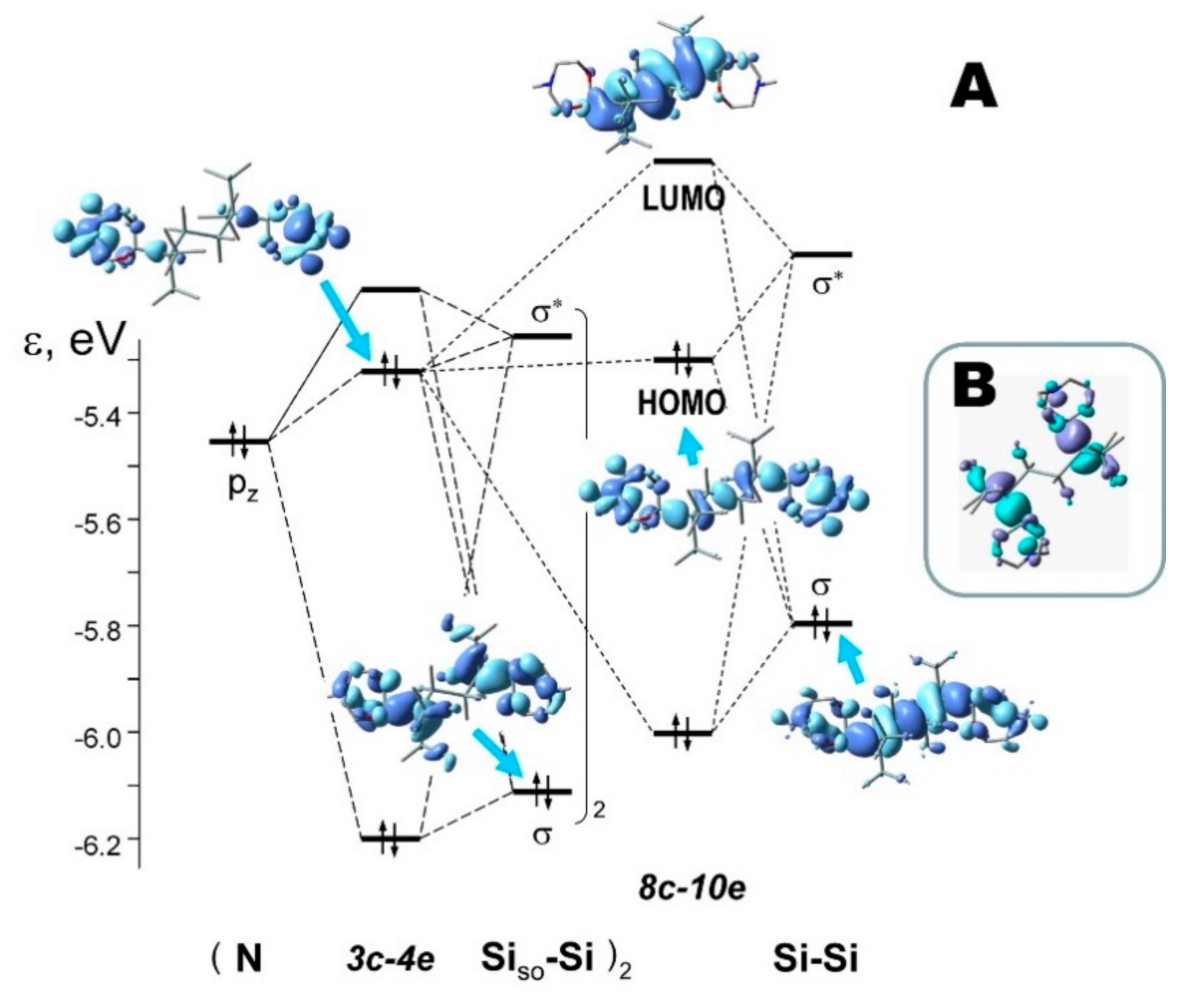
(**A**) Two 3c-4e systems of silocane units and resulting nonbonding 8c-10e HOMO in **5** from DFT B3LYP/aug-CC-pVQZ in acetonitrile (C-PCM). (**B**) For comparison, HOMO of 1,4-disilatranyltetrasilane, the Si_4_ chain has the same orientation as in (**A**).

In **5**, there are several potential sites of ET: the tetrasilane chain (oligosilanes are oxidizable at *E*_p_ ≅ 1.3–1.7 V vs. SCE) [43,44,45,46,50,51,52] and two silatranyl units. Note that a donor effect of the latter might lower the oxidation potential of the connecting Si_4_ bridge [53]. A closer look at the XRD and DFT data of **5** reveals an interesting feature of its structure directly related to the electron-releasing ability. The N→Si contacts of both silatranyl units in **5** lie in the plane of the Si_4_ chain (Figure 1), whose s-*trans* zig-zag geometry allows conjugation of these two terminal 3c-4e units via their external Si_(silocane)_-Si σ-elements (Figure 11). As a consequence, HOMO of **5** is additionally uplifted making the ionization easier so that **5** has the lowest *E*_p_ in this series. This effect can be seen as the formation of a common end-to-end multicenter-multielectron (8c–10e) system (Figure 11), the first case of this kind. Interestingly, although 3c–4e interaction is stronger in silatranes, this effect is not realized in the homologous bis-silatrane with the same tetrasilanyl chain [4] because the orientation of the two silatranyl units is perpendicular to the central Si-Si bond of the Si_4_ bridge, excluding such end-to-end conjugation (Figure 11). 

Silocane **7** has several peculiar features. Second-order NBO perturbation analysis depicts the p_z_(N)→σ*(Si-Cl) delocalization as a hybrid bond NBO_N__→Si_ = 0.963(*sp*^4.49^)_N_ + 0.27(*sp*^1.31^)_Si_ with 1.87e population. Penta-coordination of Si in **7** (2×∠O-Si-Cl_eq_ + ∠O-Si-O = 359.5 deg) and bond critical point *bcp* (3, **−**1) found by AIM analysis in the N**^…^**Si interatomic area (with ρ(r_e_) = 0.0567 e Å^−3^ and the Laplacian of electron density ∇^2^ρ(r_e_) = 0.088 e Å^−5^) complete the picture of the formation of the 3c-4e system in this silocane.

The N→Si donation in **7** aligns one Si-Cl bond to form a N**^…^**Si**^…^**Cl_ax_ axis (though not strictly linear, ∠N-Si-Cl_ax_ = 166.3 deg), while the second Si-Cl bond might in principle adopt either an “up” (s-*cis*) or a “down” (s-*trans*, intuitively preferential) orientation relative to N-CH_3_ group. The calculations reveal an, at first sight, strange feature of the structure of **7**: in spite of the eclipsed orientation of Si-Cl_eq_ and N-C_Me_ bonds, the (s-*cis*) geometry is 7.13 kcal mol^−1^ lower in energy than the noneclipsed (s-*trans*) configuration. Dihedral ∠N-Si-Cl driver analysis shows low *trans*/*cis* (3.64 kcal mol^−1^) and a rather high *cis*/*trans* (10.76 kcal mol^−1^) interconversion barriers making the s-*cis* **7** a global minimum on its potential energy surface. The NBO analysis of electron delocalizations shows that the stabilization of the eclipsed configuration comes from σ(N-C_Me_)-σ*(Si-Cl_eq_) hyperconjugation (2.13 kcal mol^−1^). This effect turns to the opposite and becomes destabilizing both upon oxidation and reduction of **7** inducing, respectively, positive charge on N in the cation radical or an additional electron density at Si (anionic) center.

**Figure 12:**
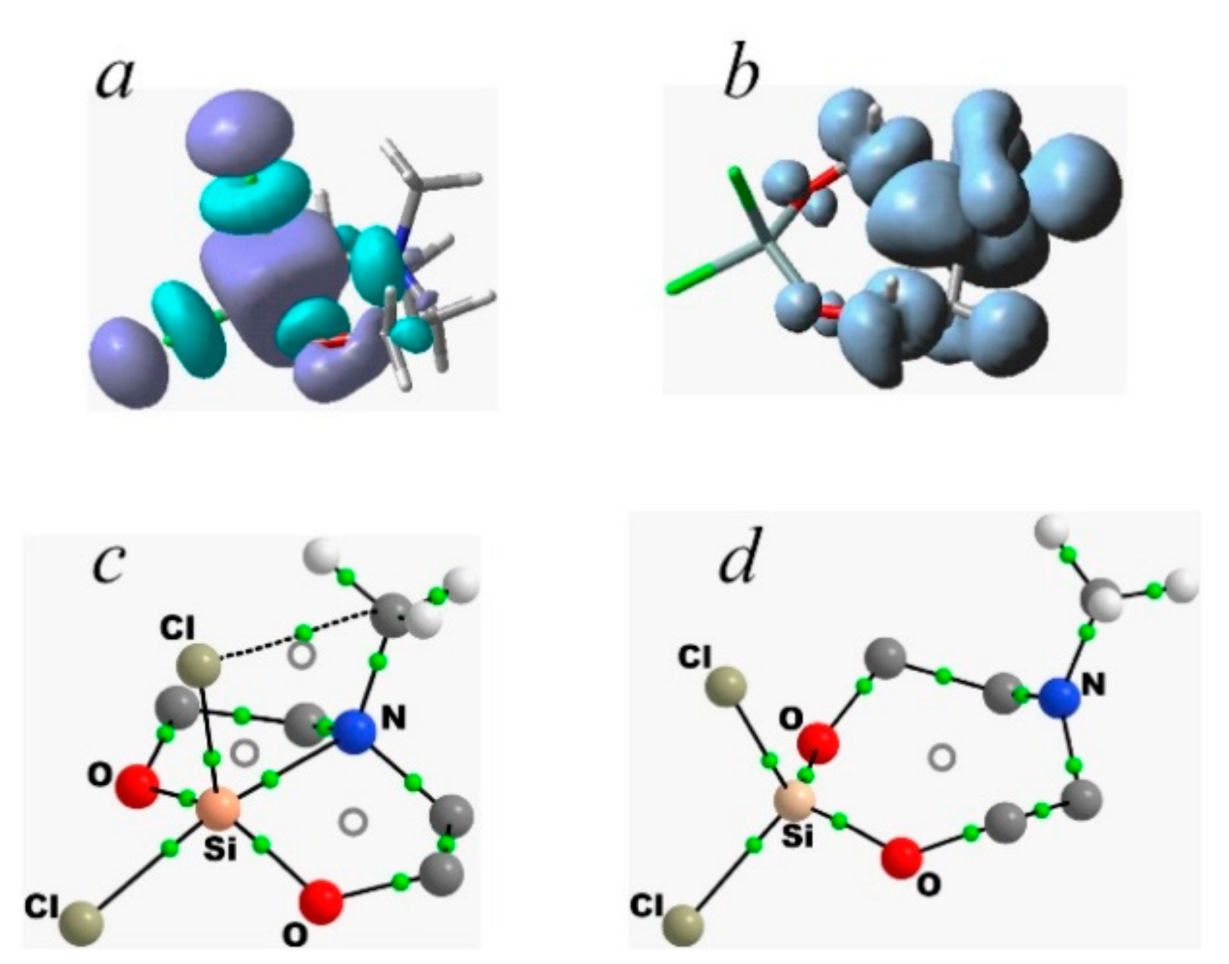
Doubly degenerated LUMO on two Si-Cl bonds of **7** (**a**) and mostly N-centered SOMO of **7**^+^^•^ (**b**). Molecular graphs of **7** (**c**) and **7**^+^^•^ (**d**) from AIM analysis; hollow circles—ring critical points *rcp* (3, +1), green circles—bond critical points *bcp* (3, −1). For clarity, only the protons of the N-Me group are shown.

AIM analysis of electron density in **7** and **7**^+^^•^ provides further insight into this feature. In the interatomic area of the C_Me_**^…^**Cl_eq_ contact, a bond-critical point *bcp* (3, −1) was found (ρ(r_e_) = 0.0097 e Å^−3^ and ∇^2^ρ(r_e_) = 0.045 e Å^−5^) showing weak but non-negligible bonding interaction. This interaction locks an additional lateral chain in this silocane, resulting in a tricyclic carcass system, a derivative of a 5-aza-1-silatricyclo[3.3.2.0]decane similar to that of silatranes (Figure 12). In line with this finding, AIM analysis reveals three ring-critical points *rcp* (3, +1) in **7** providing additional stabilization and rigidity to its structure. 

Removal of one electron from this peculiar system destroys its bonding character causing the disappearance not only of the N→Si bond critical point (bcp) (3, −1) but also of the bcp (3, −1) of the Cl**^…^**C_Me_ contact as well as of two out of three ring critical points (rcp) (3, +1): only one rcp now remains in **7**^+^^•^ (Figure 12). In fact, the sum of three ∠C-N-C angles around N atom in **7**^+^^•^ becomes 359.86 deg (332.9 deg in **7**) which depicts it as perfectly planar while the Si (5-coordinated in **7**) becomes tetrahedral (2×∠O-Si-Cl_eq_ + ∠O-Si-O = 334.6 deg, with ∠Cl-Si-Cl = 110.7 deg and ∠O-Si-O = 113.3 deg), and the ∠Cl_ax_-Si-N angle becomes 145.2. The length of the N**^…^**Si contact in **7**^+^^𢀢^ (*l*_N-Si_ = 3.649 Å) is 1.478 Å longer (68% elongation!) than in neutral **7** and is much longer than the sum of van des Waals radii of N and Si, signifying the 3c-4e system is no longer present in the oxidized state of this silocane. 

## 3. Conclusions

Extending our previous work on oligosilanylated silatranes [3,4,5,6,7], we have chosen to study the chemistry of a related system with a less constrained geometry. For this reason we decided to turn to the family of silocanes, which can be regarded as silatranes with one of the bridging arms between nitrogen and silicon missing. A potential donor acceptor interaction between nitrogen and silicon is therefore much more dependent on a very electrophilic silicon atom and has to also meet steric requirements in order to allow the nitrogen to approach the silicon atom. For this study we prepared a number of N-methyl mono- and dioligosilanylated silocanes. In none of the compounds—which include silocanes with 1-methyl-1-tris(trimethylsilyl)silyl, 1,1-bis[tris(trimethylsilyl)silyl], and 1,1-bis[tris(trimethylsilyl)germyl] substitution pattern and two examples with the silocane silicon atom being part of a cyclosilane or oxacyclosilane ring—a noticeable donor acceptor interaction from the nitrogen atom to the silicon atom was detected. The mono-tris(trimethylsilyl)silylated compound was converted to the respective silocanylbis(trimethylsilyl)silanides by previous reaction with KO*^t^*Bu. With the additional donor sites in the backbone, the compound showed some potential as a ligand for silyl lanthanide complexes [6]. Utilizing related chemistry, the cyclosilanes were transformed to oligosilane-1,3-diides by reaction with two equiv KO*^t^*Bu. Attempts to do the same with the 1,1-bis[tris(trimethylsilyl)silylated] silocane, however, led to the exchange of one tris(trimethylsilyl)silyl unit for a *tert*-butoxy substituent followed by eventual silanide formation via KO*^t^*Bu attack at the remaining tris(trimethylsilyl)silyl group. To gain more insight into the reactivity of the molecules we conducted electrochemical and computational studies on some of the compounds in addition to XRD and NMR spectroscopic characterization. The data obtained from these combined NMR-XRD-CV-DFT studies clearly show that the contact length *l*_N-Si_ in silocanes not only depends on the energy of the potential N→Si interaction but also on steric factors and fine through-space interactions of the neighboring groups at Si and N, imposing the orientation of p_z_(N) orbital relative to N**^…^**Si**^…^**X axes. This is contrasting to the case of silatranes, where a steady fixation of the N**^…^**Si**^…^**X axes by the rigid cage simplifies the situation cutting off many of the listed factors and renders *l*_N-Si_ of silatranes immediately related to the strength of N→Si donation. Therefore, comparing the extent of N→Si bonding in these classes of compounds solely on the base of *l*_N-Si_ is not quite correct and thus requires a more careful analysis.

## 4. Experimental Section

**General Remarks**. All reactions involving air-sensitive compounds were carried out under an atmosphere of dry nitrogen or argon using either Schlenk techniques or a glove box. All solvents were dried using a column-based solvent purification system [54]. Chemicals were obtained from different suppliers and used without further purification. 

MeN(CH_2_CH_2_OSiMe_3_)_2_ (**1**) [55], MeN(CH_2_CH_2_O)_2_SiMeCl (**2**),[6] tetramethyl-1,2-dichlorodisilane [56,57], 2-[tris(trimethylsilyl)silyl]-2,6-dimethyl-1,3,6,2-dioxazasilocane (**3**) [6], 1,3,-bis[tris(trimethylsilyl)silyl]-1,1,3,3-tetramethyldisiloxane [6], 1,3-bis[potassiobis(trimethylsilyl)silyl]-1,1,3,3-tetramethyldisiloxane [6], 2,2,5,5-tetrakis(trimethylsilyl)decamethylhexasilane [26,31], tetrakis(trimethylsilyl)silane [55], and tetrakis(trimethylsilyl)germane [58] were prepared following reported procedures.

^1^H (300 MHz), ^13^C (75.4 MHz), and ^29^Si (59.3 MHz) NMR spectra were recorded on a Varian INOVA 300 spectrometer. Spectra are referenced to tetramethylsilane (TMS) for ^1^H, ^13^C, and ^29^Si. Otherwise all samples were measured in CDCl_3_. To compensate for the low isotopic abundance of ^29^Si, the INEPT pulse sequence was used where possible for the amplification of the signal [59,60]. If the silocane Si signal could not be observed this way, the Varian s2pul pulse sequence (gated-decoupling) was used. Elementary analyses were carried out using a Heraeus VARIO ELEMENTAR instrument. For potassium silanides, which are very sensitive and which typically have varying amounts or solvent coordinated neither meaningful melting (decomposition) points nor elemental analyses can be obtained. 

**X-Ray Structure Determination**. For X-ray structure analyses the crystals were mounted onto the tip of glass fibers. Data collection was performed with a BRUKER-AXS SMART APEX CCD diffractometer using graphite-monochromated Mo Kα radiation (0.71073 Å). The data were reduced to F^2^_o_ and corrected for absorption effects with SAINT [61] and SADABS [62,63], respectively. The structures were solved by direct methods and refined by full-matrix least-squares method (SHELXL97) [64]. Otherwise all nonhydrogen atoms were refined with anisotropic displacement parameters. All hydrogen atoms were located in calculated positions to correspond to standard bond lengths and angles. All diagrams were drawn with 30% probability thermal ellipsoids and all hydrogen atoms were omitted for clarity. Crystallographic data (excluding structure factors) for the structures of compounds **5**, **8**, **8a**, **9**, **10**, and **12** reported in this paper have been deposited with the Cambridge Crystallographic Data Center as Supplementary publications no. CCDC-2048031 (**5**), 2048028 (**8**), 2048043 (**8a**), 2048030 (**9**), 2048029 (**10**), and 2048032 (**12**)**.** Copies of data can be obtained free of charge at: http://www.ccdc.cam.ac.uk/products/csd/request/. 

**Electrochemistry.** Cyclic- and square-wave-pulsed voltammetry was carried out using a PAR-2373 potentiostat (under Power Suite 2.58 software) [65] connected to a 5-mL, three-electrode glass cell. Either 2.5-mm glassy carbon (GC) or 0.5-mm Pt disks served as working electrodes. The potentials are referred to as Ag/AgCl reference electrode and checked relative to the Fc^+^/Fc reversible system (E_0_ = 0.40 V vs. SCE) [66] used as an internal standard for tuning the IR-correction. Square-wave-pulsed voltammetry parameters: pulse 25 mV for 30 ms, step 20 mV, *v* = 333 mV s^−1^. Vacuum-dried Bu_4_NPF_6_ (80–90 °C for 5 h) was used as a supporting electrolyte (0.1 M solutions) in acetonitrile (AN) and, when necessary for increasing the solubility of the substrates, in the mixtures AN-THF. AN and THF were distilled over CaH_2_ and sodium benzophenone ketyl, respectively, and additionally dried over activated 4 Å molecular sieves. All measurements were carried out under argon in a glovebox.

**Computational analysis.** With comparable outcome for the geometries, the quadruple correlation consistent aug-CC-pVQZ basis set was earlier found to provide better account for dative interactions and charge distribution in silocanes than Lanl2DZ [17], so the DFT B3LYP/aug-CC-pVQZ//HF/6-211G calculation scheme was used with Tomasi’s polarized continuum C-PCM model [67] of solvation in AN with scaled van der Waals cavity type as implemented in Gaussian 2003 [68]. AIM analysis [69] was run with the electron density from DFT B3LYP/aug-CC-pVQZ using AIMAll program [70].

### 4.1. Potassiobis(trimethylsilyl)silyl]-2,6-dimethyl-1,3,6,2-dioxazasilocane 18-crown-6 **(4)**

A solution of **3** (48 mg, 0.117 mmol), KO*^t^*Bu (14 mg, 0.123 mmol), and 18-crown-6 (32 mg, 0.123 mmol) in benzene (1 mL) was stirred at ambient temperature. After 14 h NMR spectroscopy confirmed complete reaction. Yellow crystalline **4** (74 mg, 100%) precipitated from the solution. NMR (δ in ppm, C_6_D_6_): ^1^H: 4.14 (m, 2H), 4.02 (m, 2H), 3.28 (s, 24H, C*H_2_*O), 2.89 (m, 2H), 2.72 (m, 2H), 2.42 (s, 3H, MeN), 0.77 (s, 3H, SiMe*_3_*), 0.71 (s, 18H, Me_3_Si). ^13^C: 70.1 (*C*H_2_O), 63.0 (O*C*H_2_), 60.7 (N*C*H_2_), 46.2 (MeN), 7.5 (Me_3_Si), 6.4 (MeSi). For ^29^Si see Ref. [6]

### 4.2. 2,2’-(2,2,3,3-Tetramethyl-1,1,4,4-tetrakis(trimethylsilyl)tetrasilane-1,4-diyl)bis(2,6-di methyl-1,3,6,2-dioxazasilocane) **(5)**

A solution of freshly prepared **4** [obtained from: **3** (221 mg, 0.542 mmol) and KO*^t^*Bu (62 mg, 0.558 mmol)] in toluene (3 mL) was added dropwise over 15 min to an ice-cooled solution of 1,2-dichlorotetramethyldisilane (56 mg, 0.298 mmol) in toluene (3 mL). After 12 h the volatiles were removed in vacuo and the residue was dissolved in benzene and filtered. After crystallization from diethylether and acetonitrile (2:1) colorless crystalline **5** (150 mg, 70%) was obtained. Mp: 128–137 °C. NMR (δ in ppm in CDCl_3_): ^1^H: 3.70 (m, 8H), 2.57 (t, *J* = 4.4 Hz, 8H), 2.41 (s, 6H, MeN), 0.36 (s, 12H, SiMe_2_), 0.34 (s, 6H, SiMe), 0.22 (s, 36H, M_3_Si). ^13^C: 62.32 (OCH_2_), 58.65 (NCH_2_), 43.91 (MeN), 3.63 (MeSi), 3.11 (Me_3_Si), 0.18 (Me_2_Si). ^29^Si: 2.5 (SiO_2_), −9.6 (Me_3_Si), −31.0 (Me_2_Si), −129.6 (Si_q_). Anal. Calcd. For C_28_H_76_Si_10_O_4_N_2_ (785.78): C 42.80, H 9.75, N 3.57. Found: C 42.45, H 9.41, N 3.43. UV absorption: λ = 254 nm (ε = 7.42 × 10^4^ M^−1^cm^−1^) in *n*-hexane.

### 4.3. 2,2-Dimethoxy-6-methyl-1,3,6,2-dioxazasilocane **(6)**

A solution of N-methyldiethanolamine (1.000 g, 8.390 mmol), tetramethoxysilane (1.405 g, 9.230 mmol), and tetraethylammonium fluoride hydrate (31 mg, 0.208 mmol) in toluene (10 mL) was heated to reflux for 12 h. After removal of the volatiles, compound **6** was obtained as a colorless oil (1.685 g, 97%). NMR (δ in ppm, C_6_D_6_): ^1^H: 3.67 (s, 6H, OMe), 3.52 (m, 4H), 2.68 (m, 1H), 2.26 (m, 1H), 2.09 (m, 2H), 2.04 (s, 3H, NMe). ^13^C: 60.2 (OCH_2_), 55.3 (NCH_2_), 51.5 (SiOMe), 43.1 (MeN). ^29^Si: −90.7.

### 4.4. 2.2-Dichloro-6-methyl-1,3,6,2-dioxazasilocane **(7)**

To a solution of **6** (1.68 g, 8.13 mmol) in toluene (5 mL) thionyl chloride (4 mL) was added dropwise over 30 min and stirring was continued for 14 h. By decantation a pale green residue was obtained. The residue was mixed with toluene (5 mL) and thionyl chloride (5 mL) and again decanted. After removal of the volatiles in vacuo, a pale-green-orange powder of **7** (1.72 g, 98%) was obtained. NMR (δ in ppm): ^1^H (at 0 °C): 4.03 (t, *J* = 6.0 Hz, 4H, OCH_2_), 3.08 (quintet, *J* = 6.0 Hz, 2H), 2.92 (quintet, *J* = 6.0 Hz, 2H), 2.68 (s, 3H, MeN). ^13^C: 58.9 (OCH_2_), 53.8 (NCH_2_), 44.9 (MeN). ^29^Si: −89.4.

### 4.5. 2,2-Bis[tris(trimethylsilyl)silyl]-6-methyl-1,3,6,2-dioxazasilocane **(8)**

A solution of tetrakis(trimethylsilyl)silane (200 mg, 0.624 mmol) and KO*^t^*Bu (72 mg, 0.642 mmol) dissolved in THF (3 mL) was stirred at ambient temperature. After NMR spectroscopy confirmed complete conversion, the solvent was removed, the residue dissolved in toluene (5 mL) and quickly added to **7** (114 mg, 0.530 mmol) in toluene (2 mL). After 6 h volatiles were removed, the residue dissolved in benzene and filtered. Colorless crystals of **8** (132 mg, 66%) were obtained from a mixture of diethylether and acetonitrile 2:1. Mp: 192-208 °C. NMR (δ in ppm): ^1^H: 3.82 (t, *J* = 4.6 Hz, 4H, OCH_2_), 2.66 (t, *J* = 4.7 Hz, 4H, NCH_2_), 2.37 (s, 3H, MeN), 0.28 (s, 54H, (C*H_3_*)_3_Si). ^13^C: 66.4 (OCH_2_), 59.3 (NCH_2_), 47.3 (MeN), 4.0 (Me*_3_*Si). ^29^Si: 19.6 (SiO_2_), −9.6 (Me_3_Si), −121.0 (Si_q_). Anal. Calcd. For C_23_H_65_NO_2_Si_9_ (640.54): C 43.13, H 10.23, N 2.19. Found: C 43.89, H 8.62, N 2.12. UV absorption: λ = 260 nm (ε = 2.54 × 10^4^ M^−1^cm^−1^) in diethylether.

### 4.6. 2,2-Bis[tris(trimethylsilyl)germyl]-6-methyl-1,3,6,2-dioxazasilocane **(8a)**

The reaction was done according to the procedure for **8** using: tetrakis(trimethylsilyl)germane (400 mg, 1.095 mmol), KO*^t^*Bu (126 mg, 1.128 mmol), and **7** (200 mg, 0.925 mmol). For purification the compound was subjected to sublimation at 45 °C/0 mbar to remove tetrakis(trimethylsilyl)germane. Colorless crystals of **8a** (153 mg, 38%) were obtained from diethylether and acetonitrile 2:1. Mp: 139-148 °C. NMR (δ in ppm): ^1^H: 3.81 (t, *J* = 4.4 Hz, 4H, OCH_2_), 2.66 (t, *J* = 4.4 Hz, 4H, NCH_2_), 2.37 (s, 3H, MeN), 0.31 (s, 54H, Me_3_Si). ^13^C: 66.1 (OCH_2_), 59.3 (NCH_2_), 47.4 (MeN), 4.4 (Me_3_Si). ^29^Si: 17.7 (SiO_2_), −4.6 (Me_3_Si). Anal. Calcd. For C_23_H_65_Ge_2_NO_2_Si_7_ (729.63): C 37.86, H 8.98, N 1.92. Found: C 38.66, H 7.70, N 1.86. UV absorption: λ = 256 nm (ε = 4.18 × 10^4^ M^−1^cm^−1^) in diethylether.

### 4.7. 2,2,3,3,9-Pentamethyl-1,1,4,4-tetrakis(trimethylsilyl)-6,12-dioxa-9-aza-1,2,3,4,5-penta silaspiro[4.7]dodecane **(9)**

2,2,5,5-Tetrakis(trimethylsilyl)decamethylhexasilane (200 mg, 0.327 mmol) and KO*^t^*Bu (77 mg, 0.686 mmol) were dissolved in THF (5 mL). After NMR spectroscopy confirmed formation of oligosilyldipotassium, the THF was removed, the residue dissolved in toluene (5 mL) and then added a solution of **7** (73 mg (0.336 mmol, 1.03) in toluene (2 mL). After 21 h the volatiles were removed and the residue was extracted with pentane (2 × 2 mL), centrifuged and filtered. Colorless crystals of **9** (152 mg, 90%) were obtained from a mixture of acetonitrile and diethylether 1:2. Mp: 242-250 °C. NMR (δ in ppm): ^1^H: 3.82 (t, *J* = 4.6 Hz, 4H, OCH_2_), 2.67 (t, *J* = 4.6 Hz, 4H, NCH_2_), 2.46 (s, 3H, MeN), 0.31 (s, 12, SiMe_2_), 0.24 (s, 36H, SiMe_3_). ^13^C: 65.1 (OCH_2_), 58.2 (NCH_2_), 45.9 (MeN), 3.1 (Me_3_Si), −2.2 (Me_2_Si). ^29^Si: 33.1 (SiO_2_), −7.7 (Me_3_Si), −30.4 (Me_2_Si), −133.2 (Si_q_). Anal. Calcd. For C_21_H_59_NO_2_Si_9_ (610.47): C 41.32, H 9.74, N 2.29. Found: C 39.16, H 9.76, N 1.95. UV absorption: λ_1_ = 241 nm (ε = 2.23 × 10^4^ M^−1^cm^−1^), λ_2_ = 255 nm (ε = 1.96 × 10^4^ M^−1^cm^−1^) in diethylether.

### 4.8. 2,2,4,4,10-Pentamethyl-1,1,5,5-tetrakis(trimethylsilyl)-3,7,13-trioxa-10-aza-1,2,4,5,6-penta silaspiro[5.7]tridecane **(10)**

1,3,-Bis[tris(trimethylsilyl)silyl]-1,1,3,3-tetramethyldisiloxane (687 mg, 1.10 mmol) and KO*^t^*Bu (258 mg, 2.30 mmol) were dissolved in THF (6 mL) and were kept at rt for 25 h until NMR analysis showed full conversion toward 1,3-bis[potassiobis(trimethylsilyl)silyl]-1,1,3,3-tetramethyldisiloxane. Volatiles were removed under reduced pressure and toluene (4 mL) was added to give a yellow solution. The dissolved disilanide was added within 10 min to a slurry of **7** (253 mg, 1.17 mmol) in toluene (4 mL) and was stirred for 19 h at rt. Solvents were removed under reduced pressure. The now greenish yellow residue was extracted with pentane (3 × 3 mL). After centrifugation and filtration, yellow crystals of **10** (200 mg, 29%) and an impure orange oil (360 mg, 53%) were obtained. Mp.: 162-164 °C. NMR (δ in ppm, C_6_D_6_): ^1^H: 3.75 (m, 4H), 2.31 (m, 4H), 2.18 (s, 3H), 0.54 (s, 12H), 0.43 (s, 36H). ^13^C: 66.0, 58.6, 47.1, 8.0, 3.5. ^29^Si: 19.2 (s, SiO_2_), 12.5 (s, SiMe_2_), −9.5 (s, SiMe_3_), −131.8 (s, Si*_q_*).

### 4.9. 2.-[Potassiobis(trimethylsilyl)silyl]-2-tert-butoxy-6-methyl-1,3,6,2-dioxazasilocane **(12)**

A solution of silocane **8** (154 mg, 0.240 mmol) and KO*^t^*Bu (54 mg, 0.4812 mmol) in benzene (3 mL) was heated to 70 °C for 7 d. The reaction was monitored using NMR analysis during this period, until full conversion toward silanide **12** and tris(trimethylsilyl)silyl potassium was reached. Bright-yellow crystals of **12** (80 mg, 78%) were obtained directly from the reaction solution upon cooling to rt. NMR (δ in ppm, d^8^-THF): ^1^H: 3.81 (m, 4H), 2.55 (m, 4H), 2.38 (s, 3H), 1.35 (s, 9H), 0.09 (s, 18H). ^13^C: 72.5, 62.8, 61.2, 48.1, 33.2, 7.0. ^29^Si: −5.4 (s, SiMe_3_), −13.0 (s, SiO_3_), −208.7 (s, Si*_q_*).

A solution of silocane **8** (58 mg, 0.091 mmol), 18-crown-6 (51 mg, 0.19 mmol,), and KO*^t^*Bu (21 mg, 0.19 mmol.) in benzene (1 mL) was left for reaction at rt for 20 h. The reaction was monitored with NMR analysis during this period, until full conversion toward silanide **12** and tris(trimethylsilyl)silyl potassium was detected. No separation between tris(trimethylsilyl)silyl potassium 18-crown-6 and **12** 18-crown-6 was possible.

### 4.10. 2.-[Potassiobis(trimethylsilyl)silyl]-2-[tris(trimethylsilyl)silyl]-6-methyl-1,3,6,2-dioxaza silocane **(13)**

A solution of tetrakis(trimethylsilyl)silane (32 mg, 0.10 mmol) and KO*^t^*Bu (12 mg, 0.11 mmol) in DME (1 mL) was left for reaction at ambient temperature for 22 h. Full conversion to tris(trimethylsilyl)silyl potassium and no excess of KO*^t^*Bu was detected by NMR analysis. Silocane **8** (31 mg, 0.05 mmol) was added and the reaction was left for further reaction at rt. Reaction progress was monitored by NMR analysis. The reaction was found to stop at the monoanionic silocane **13**. Attempts to separate **13** from tris(trimethylsilyl)silyl potassium and tetrakis(trimethylsilyl)silane were not successful**.** NMR (δ in ppm, D_2_O capillary in DME): ^29^Si: −5.1 (s, (Me_3_*Si*)_2_SiK), −10.1 (s, (Me_3_*Si*)_3_Si), −132.0 (s, (Me_3_Si)_3_*Si*), −182.3 (s, (Me_3_Si)_2_*Si*K).

### 4.11. 2,2,3,3,9-Pentamethyl-1,4-dipotassio-1,4-bis(trimethylsilyl)-6,12-dioxa-9-aza 1,2,3,4,5-pentasilaspiro[4.7]dodecane **(14)**

A solution of **9** (54 mg, 0.088 mmol) and KO*^t^*Bu (20 mg, 0.178 mmolin benzene (2 mL) was left at 60 °C for 17 h. After cooling to rt, yellow crystals of **14** formed in a bright-yellow solution. The crystals (47 mg, >95%) were decanted and dried under reduced pressure**.** NMR (δ in ppm, d^8^-THF): ^1^H: 3.86 (m, 4H), 2.54 (m, 4H), 2.37 (s, 3H), 0.14 (s, 12H), 0.06 (s, 18H). ^13^C: 64.8, 60.6, 47.3, 6.7, 2.2. ^29^Si: 91.5 (s, SiO_2_), −9.0 (s, SiMe_3_), −17.7 (s, SiMe_2_), −182.9 (s, Si*_q_*).

### 4.12. 2,2,4,4,10-Pentamethyl-1,5-dipotassio-1,5-bis(trimethylsilyl)-3,7,13-trioxa-10-aza 1,2,4,5,6-pentasilaspiro[5.7]tridecane **(15)**

As solution of **10** (129 mg, 0.206 mmol) and KO*^t^*Bu (48 mg, 0.428 mmol) in benzene (3 mL) and was allowed to react at 70 °C. Within 20 min the initially yellow solution turned orange and after 20 h slightly orange crystals of 15 (50 mg, 44%) were obtained**.** NMR (δ in ppm, d^8^-THF): ^1^H: 3.90 (t, *J* = 4.5 Hz, 4H), 3.61 (t, *J* = 6.1 Hz, 4H), 2.57 (t, *J* = 4.6 Hz, 4H), 2.38 (s, 3H), 1.77 (quint., *J* = 3.1 Hz, 4H), 0.14 (s, 12H), 0.09 (s, 18H). ^13^C: 68.4, 64.5, 61.7, 47.9, 26.5, 11.6, 7.7. ^29^Si: 74.0 (s, SiO_2_), 22.5 (s, SiMe_2_), −10.2 (s, SiMe_3_), −180.9 (s, Si*_q_*).

## Figures and Tables

**Table 1:** ^13^C and ^29^Si NMR properties.

Compd	C^1^ (N-Me)	C^2^ (CH_2_-N)	C^3^ (CH_2_-O)	C^4^ (Si-Me)	Si^1^ (SiO_2_)	Si^2^ (Si_q_)	Si^3^ SiMe_3_	Si^4^
**1**	43.6	54.6	59.7	3.9	−56.3	n.a.	n.a.	n.a.
**3 ^a, d^**	44.3	58.3	62.3	3.7	3.5	−134.8	−10.1	n.a.
**4 ^b^**	46.2	60.7	63.0	6.4	35.9	−210.2	−5.4	n.a.
**5 ^a^**	43.9	58.7	62.3	3.6	2.5	−129.6	−9.6	−31.0
**6 ^b^**	43.1	55.3	60.2	n.a.	−90.7	n.a.	n.a.	n.a.
**7 ^a^**	44.9	53.8	58.9	n.a.	−89.4	n.a.	n.a.	n.a.
**8 ^a^**	47.3	59.3	66.4	n.a.	19.6	−121.0	−9.6	n.a.
**8a ^a^**	47.4	59.3	66.1	n.a.	17.7	n.a.	−4.6	n.a.
**9 ^a^**	45.9	58.2	65.1	n.a.	33.1	−133.2.	−7.7	−30.4
**10 ^b^**	47.1	58.6	66.0	n.a.	19.2	−131.8	−9.5	12.5
**12 ^c^**	48.1	61.2	62.8	n.a.	−13.0	−208.7	−5.4	n.a.
**14 ^c^**	47.3	60.6	64.8	n.a.	91.5	−182.9	−9.0	−17.7
**15 ^c^**	47.9	61.7	64.5	n.a.	74.0	−180.9	−10.2	22.5

^a^ measured in CDCl_3._^b^ measured in C_6_D_6_. ^c^ measured in THF-d_8_. ^d^ taken from ref. [6].

**Table 2:** Parameters of electrochemical oxidation of silocanes (GC electrode, AN/0.1 M Bu_4_NPF_6_, *v* = 0.1 V s^−1^).

Compd	*l*_N…Si_, Å	*E*_p_, V	*E*_p-p/2_, mV	*E*_p_^a^−*E*_p_^c^, mV	n	*k*_s_, cm s^−1^
**5**	2.934	1.173	75	85	1.0	0.03
**7**	2.171 ^a^	1.325	104	250	1.0	1.3 × 10^−3^
**8**	3.624	1.345	90		1.0	0.7 × 10^−2^
**9**	3.425	1.365	81	210	0.9	

^a^ From DFT B3LYP/aug-CC-pVQZ (C-PCM, ε = 37).

## Data Availability

The data presented in this study are available in Supplementary Materials.

## Supplementary Materials

The following are available online, tabulated crystallographic data for compounds **5**, **8**, **8a**, **9**, **10**, and **12** and ^1^H, ^13^C, and ^29^Si, NMR spectra of compounds **4**, **5**, **7**, **8**, **8a**, **9**, **10**, **12**, **13**, **14**, and **15**.
