# Supplementary material for: Oligosilanylated Silocanes
Source: Molecules. 2021 Jan 5;26(1):244. doi: 10.3390/molecules26010244 (PMC7796475; doi:10.3390/molecules26010244)
Supplement: Supplementary file 1 [file molecules-26-00244-s001.pdf]

Oligosilanylated Silocanes †

Mohammad Aghazadeh Meshgi [1], Alexander Pöcheim [1], Judith Baumgartner [1,*], Viatcheslav V. Jouikov [2,*] and Christoph Marschner [1,*]

[1] Institut für Anorganische Chemie, Technische Universität Graz, Stremayrgasse 9, A-8010 Graz, Austria; agmineralag@yahoo.com (M.A.M.); alexander.poecheim@tugraz.at (A.P.)
[2] UMR 6226—ISCR, Université de Rennes 1, 35042 Rennes, France
* Correspondence: baumgartner@tugraz.at (J.B.); viatcheslav.jouikov@univ-rennes1.fr (V.V.J.); christoph.marschner@tugraz.at (C.M.)
† Dedicated to Professor Josef Michl on the occasion of his 80th birthday and in recognition of his numerous exciting contributions to organosilicon chemistry.

Contents

1. Crystallographic table of compounds **5**, **8**, **8a**, **9**, **10** and **12**
2. NMR spectra

Compound **4:** ^{1}H, ^{13}C and ^{29}Si INEPT NMR spectra
Compound **5:** ^{1}H, ^{13}C and ^{29}Si INEPT NMR spectra
Compound **7:** ^{1}H, ^{13}C and ^{29}Si inversed gated NMR spectra
Compound **8:** ^{1}H, ^{13}C and ^{29}Si inversed gated NMR spectra
Compound **8a:** ^{1}H, ^{13}C and ^{29}Si inversed gated NMR spectra
Compound **9:** ^{1}H, ^{13}C and ^{29}Si INEPT NMR spectra
Compound **10:** ^{1}H, ^{13}C and ^{29}Si inversed gated NMR spectra
Compound **12:** ^{1}H, ^{13}C and ^{29}Si inversed gated NMR spectra
Compound **13:** ^{29}Si INEPT NMR spectrum, reaction mixture
Compound **14:** ^{1}H, ^{13}C and ^{29}Si inversed gated NMR spectra
Compound **15:** ^{1}H, ^{13}C and ^{29}Si inversed gated NMR spectra

Table S1. Crystallographic data for compounds 5, 8, 8a, 9, 10 and 12.

	**5**	**8**	**8a**	**9**	**10**	**12**
Empirical formula	$C_{28}H_{76}N_2O_4Si_{10}$	$C_{23}H_{65}NO_2Si_9$	$C_{23}H_{65}NO_2Si_7$	$C_{21}H_{59}NO_2Si_9$	$C_{21}H_{59}NO_3Si_9$	$C_{15}H_{38}KNO_3Si_4$
M_w	785.81	640.57	729.57	610.50	626.50	431.92
Temperature [K]	100(2)	100(2)	100(2)	100(2)	100(2)	100(2)
Size [mm]	0.33×0.23×0.18	0.34×0.26×0.20	0.34×0.22×0.17	0.24×0.22×0.09	0.40×0.22×0.20	431.92
Crystal system	monoclinic	orthorhombic	triclinic	orthorhombic	monoclinic	Monoclinic
Space group	P2(1)/c	Pccn	P-1	Pca2(1)	P2(1)/n	P2(1)/c
a [Å]	9.773(4)	43.341(8)	9.952(2)	20.122(5)	9.879(2)	9.762(2)
b [Å]	18.507(4)	12.833(2)	12.433(2)	15.271(4)	18.655(4)	19.313(4)
c [Å]	13.604(3)	14.389(3)	17.836(3)	12.449(3)	21.111(4)	13.327(3)
α [°]	90	90	101.476(3)	90	90	90
β [°]	103.801(3)	90	92.746(3)	90	96.358(3)	100.070(4)
γ [°]	90	90	110.105(3)	90	90	90
V [Å ^{3}]	2389(2)	8003(2)	2015(2)	3825(2)	3867(2)	2473.9(9)
Z	2	8	2	4	4	4
ρ_{calc} [gcm^{-3}]	1.092	1.063	1.203	1.060	1.076	1.160
Absorption coefficient [mm^{-1}]	0.305	0.318	1.719	0.330	0.330	0.421
F(000)	860	2816	776	1336	1368	936
θ range	1.89<θ<26.35	0.94<θ<26.37	1.79<θ<26.36	1.67<θ<26.38	1.46<θ<26.37	1.88<θ<26.37
Reflections collected/unique	18794/4868	55175/8159	16140/8103	29445/7810	30339/7917	19491/5050
Completeness to θ [%]	99.9	99.9	98.4	100	100	99.8
Data/restraints/parameters	4868/0/209	8159/0/335	8103/0/335	7810/0/316	7917/0/319	5050/3/269

Goodness of fit on F^2	1.14	1.11	1.05	1.13	1.25	1.26
Final R indices [$I>2\sigma(I)$]	R1=0.049	R1=0.072	R1=0.025	R1=0.046	R1=0.075	R1=0.065
	*w*R2=0.122	*w*R2=0.180	*w*R2=0.066	*w*R2=0.097	*w*R2=0.148	*w*R2=0.145
R indices (all data)	R1=0.053	R1=0.078	R1=0.029	R1=0.049	R1=0.077	R1=0.070
	*w*R2=0.125	*w*R2=0.184	*w*R2=0.068	*w*R2=0.099	*w*R2=0.149	*w*R2=0.148
Largest diff. Peak/hole [e^-/ Å^3]	0.86/-0.30	2.97/-0.54	0.67/-0.31	0.51/-0.23	0.77/-0.76	0.67/-0.28

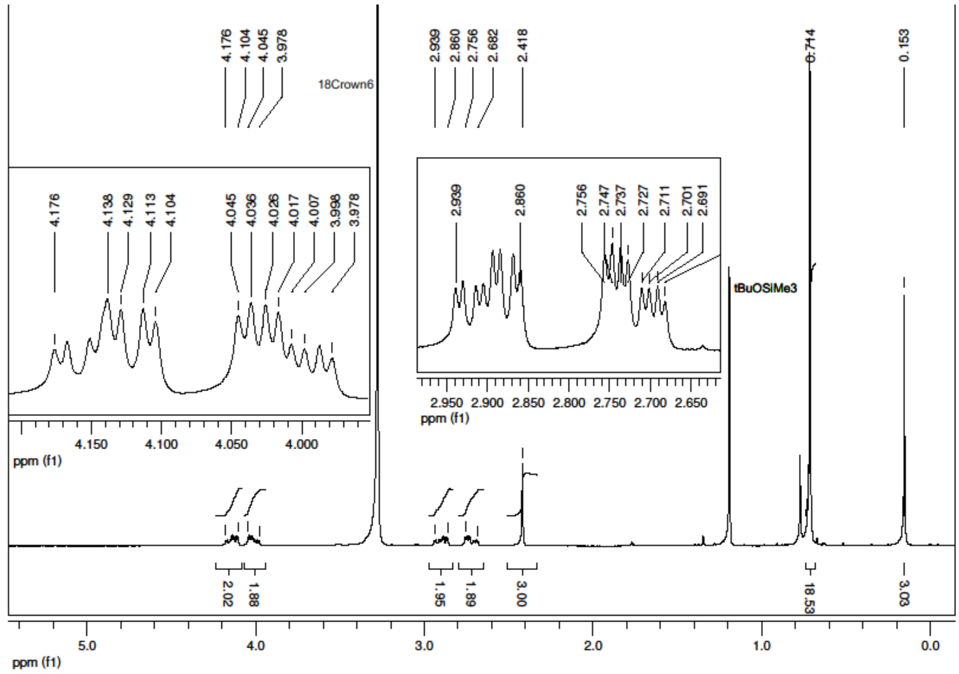

Figure S1. ^{1}H NMR spectrum of **4** in C_6C_6.

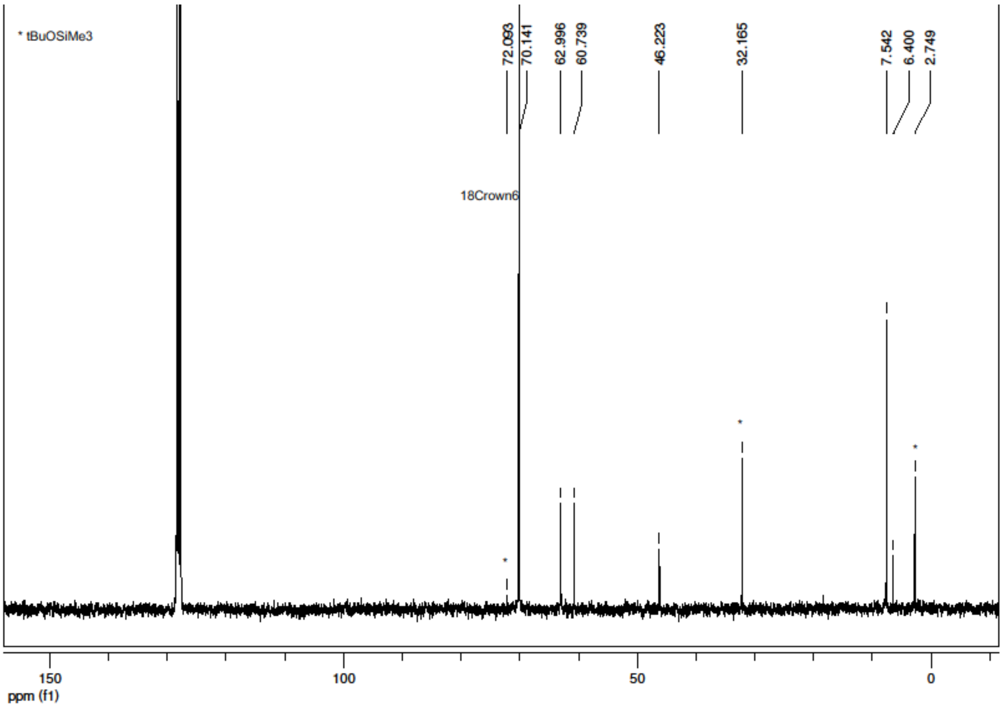

Figure S2. ^{13}C NMR spectrum of **4** in C_6C_6.

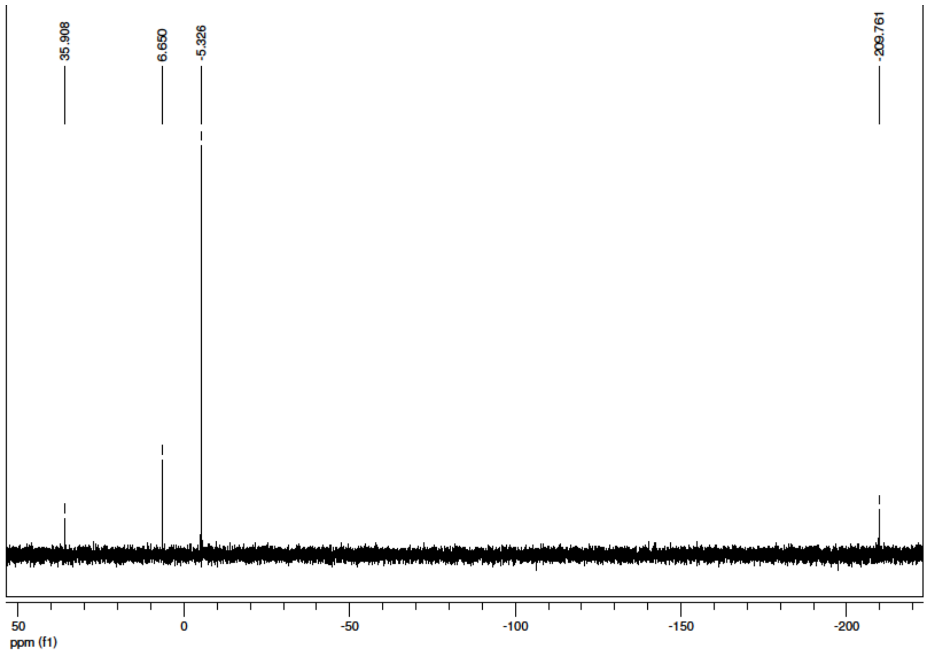

Figure S3. ^{29}Si INEPT NMR spectrum of **4** in C_6C_6.

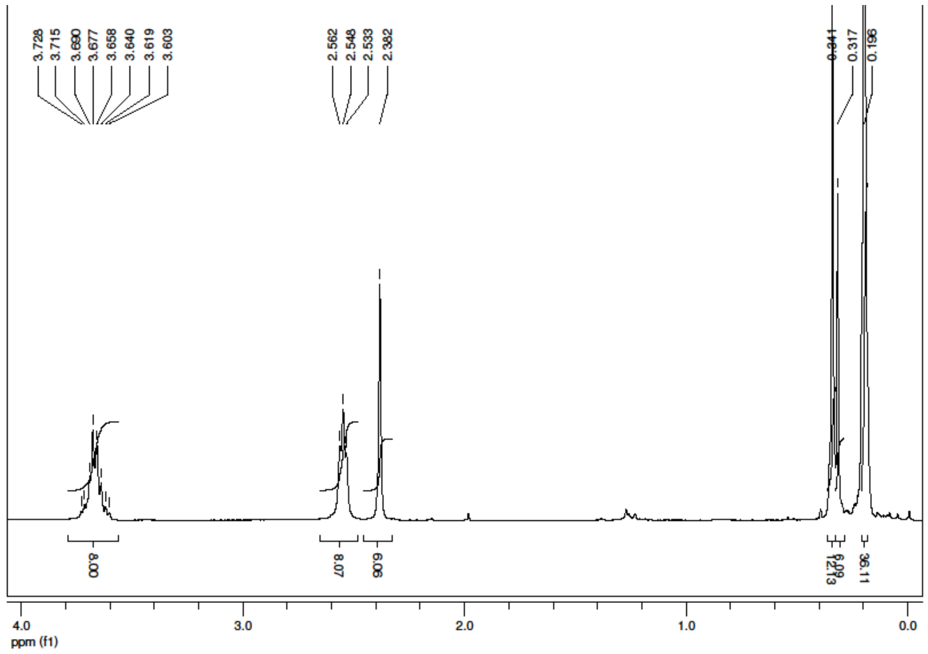

Figure S4. ^{1}H NMR spectrum of **5** in $CDCl_3$.

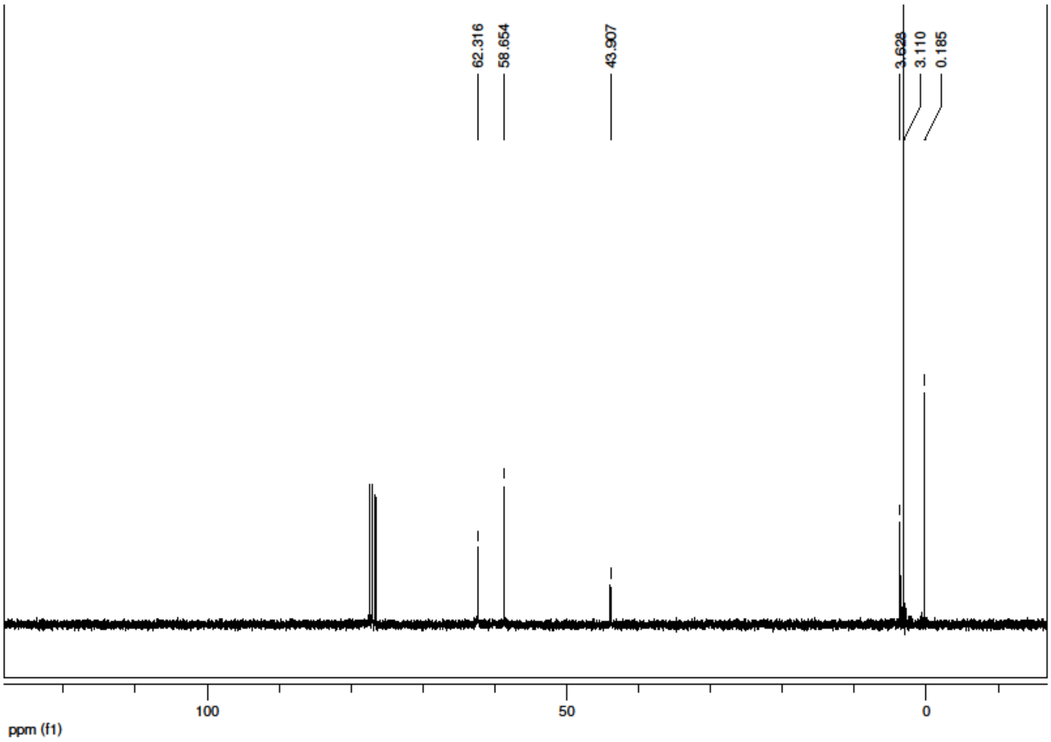

Figure S5. ^{13}C NMR spectrum of **5** in $CDCl_3$.

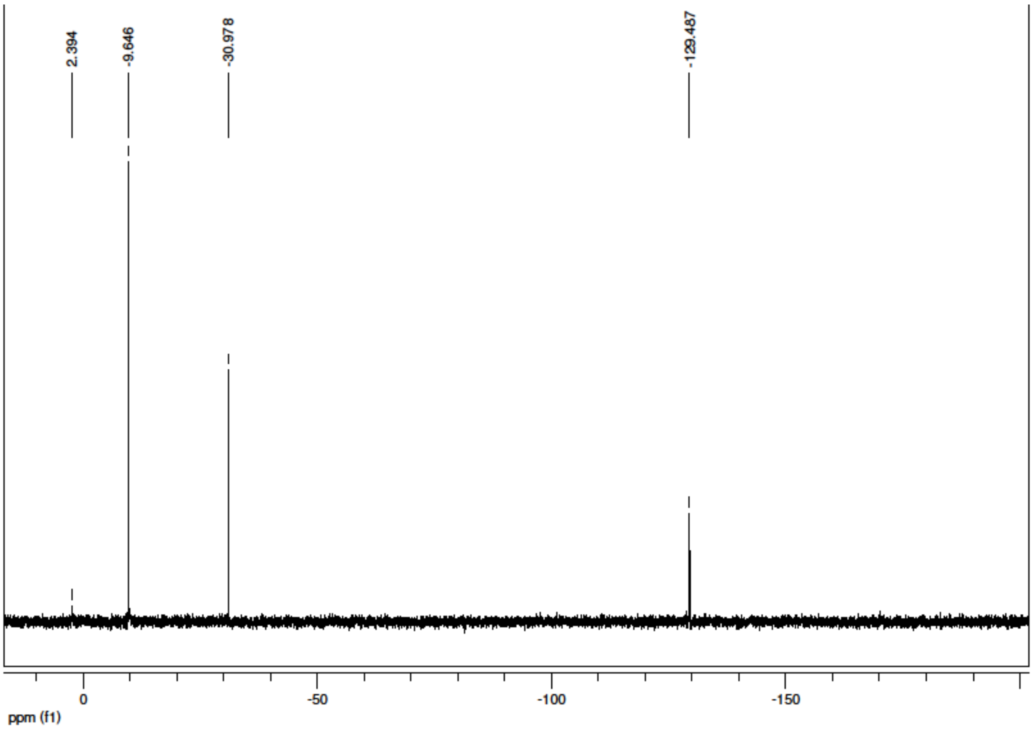

Figure S6. ^{29}Si INEPT NMR spectrum of **5** in $CDCl_3$.

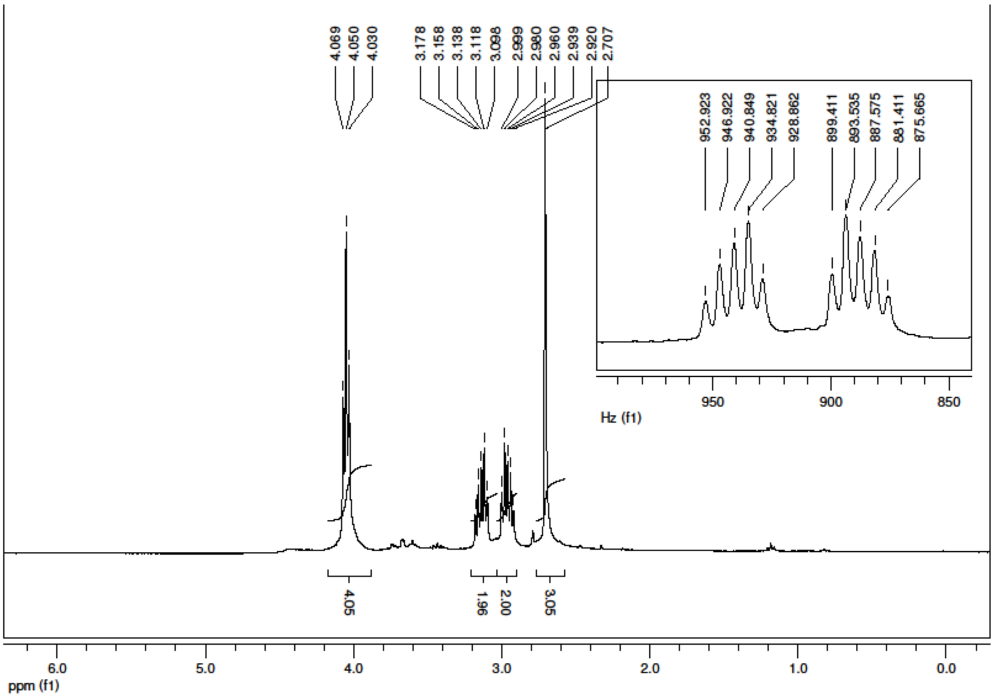

Figure S7. ^{1}H NMR spectrum of **7** at -40°C in $CDCl_3$.

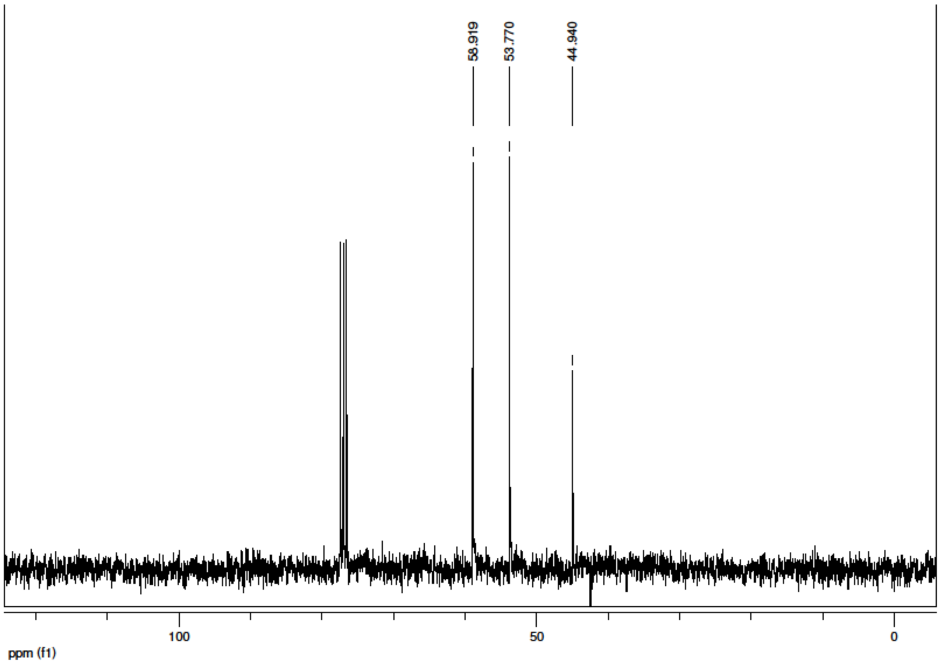

Figure S8. ^{13}C NMR spectrum of **7** in $CDCl_3$.

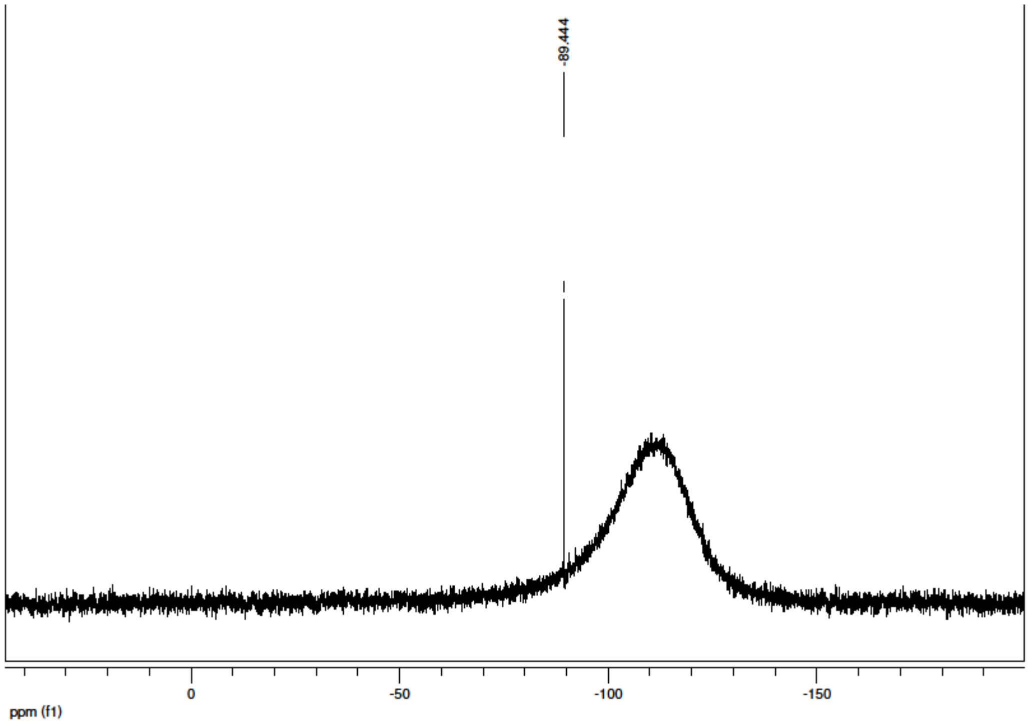

Figure S9. ^{29}Si inversed gated NMR spectrum of **7** in $CDCl_3$.

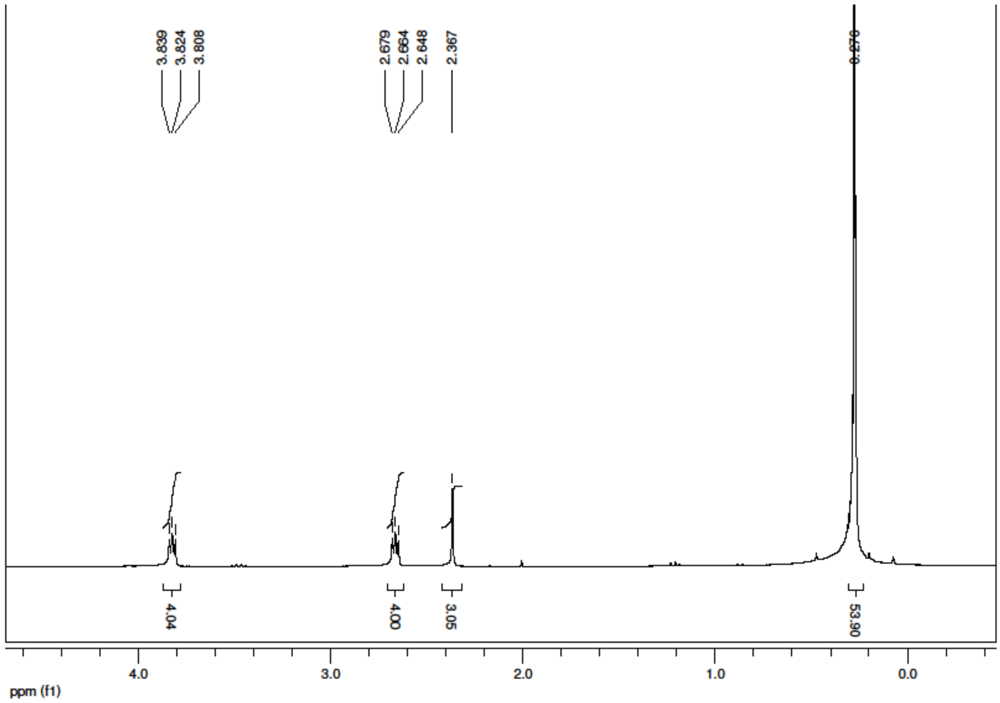

Figure S10. ^{1}H NMR spectrum of **8** in $CDCl_3$.

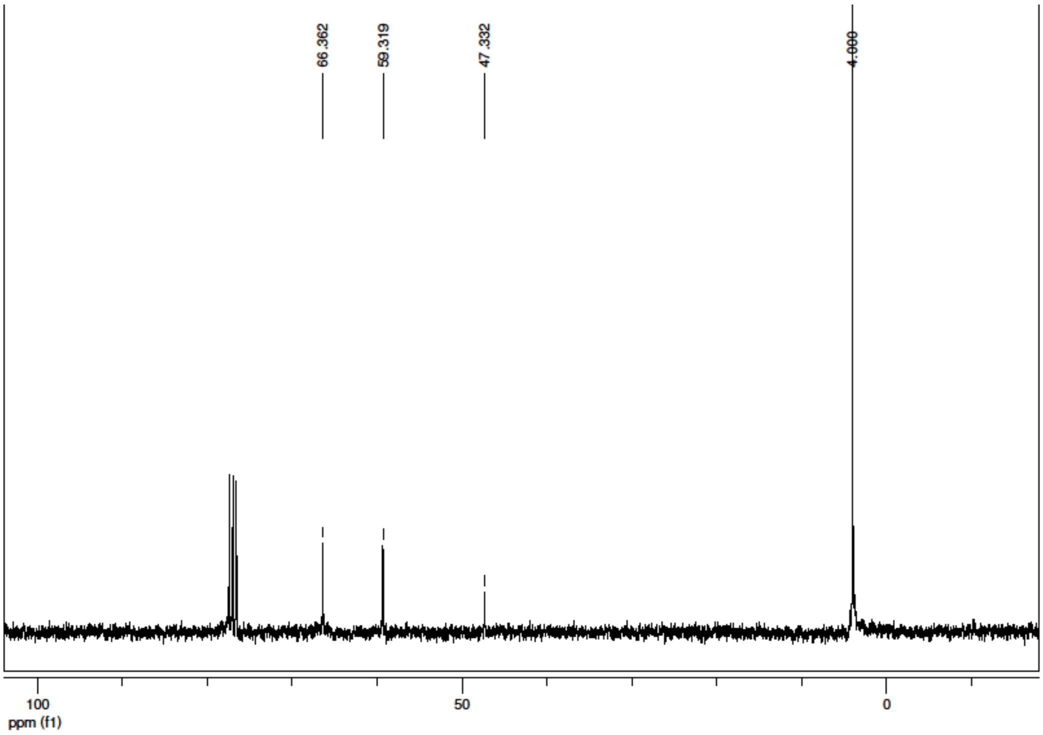

Figure S11. ^{13}C NMR spectrum of **8** in $CDCl_3$.

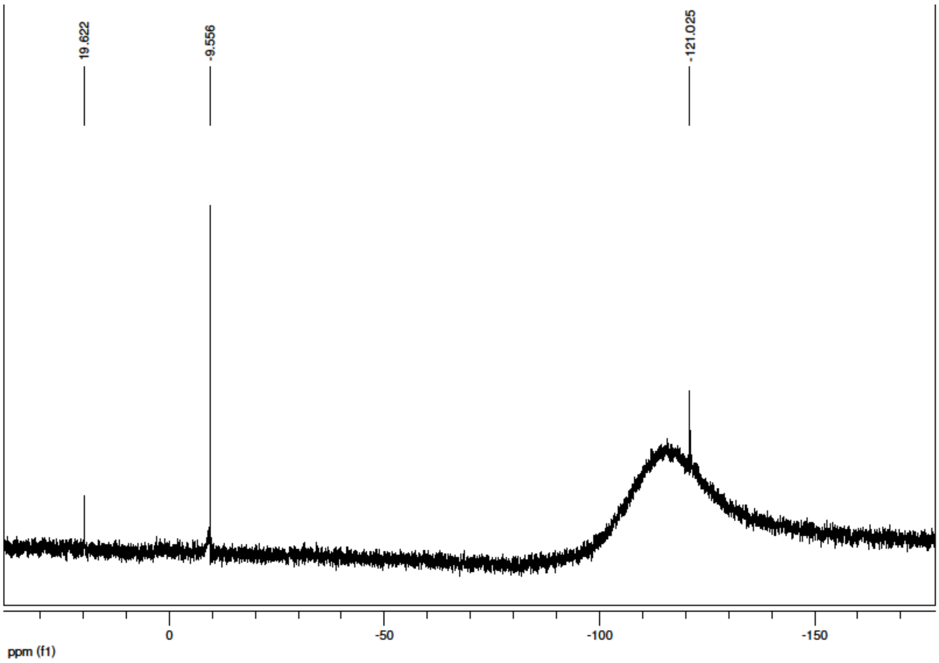

Figure S12. ^{29}Si inversed gated NMR spectrum of **8** in $CDCl_3$.

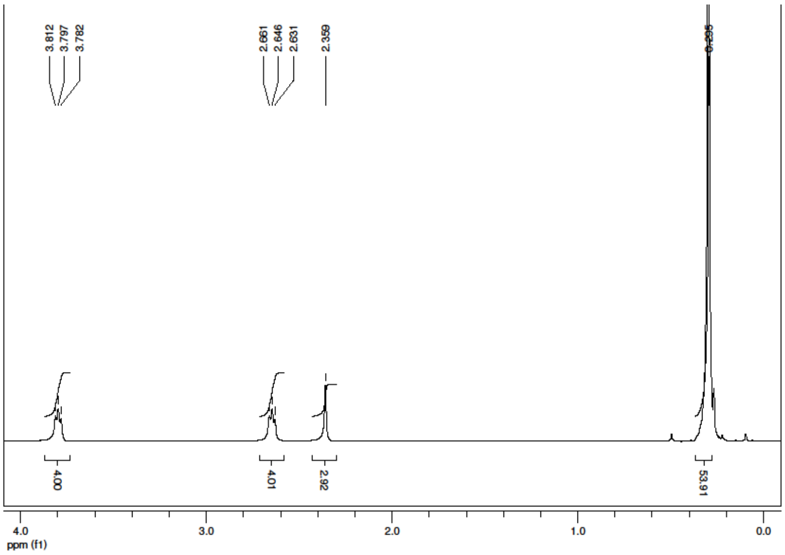

Figure S13. ^{1}H NMR spectrum of **8a** in $CDCl_3$.

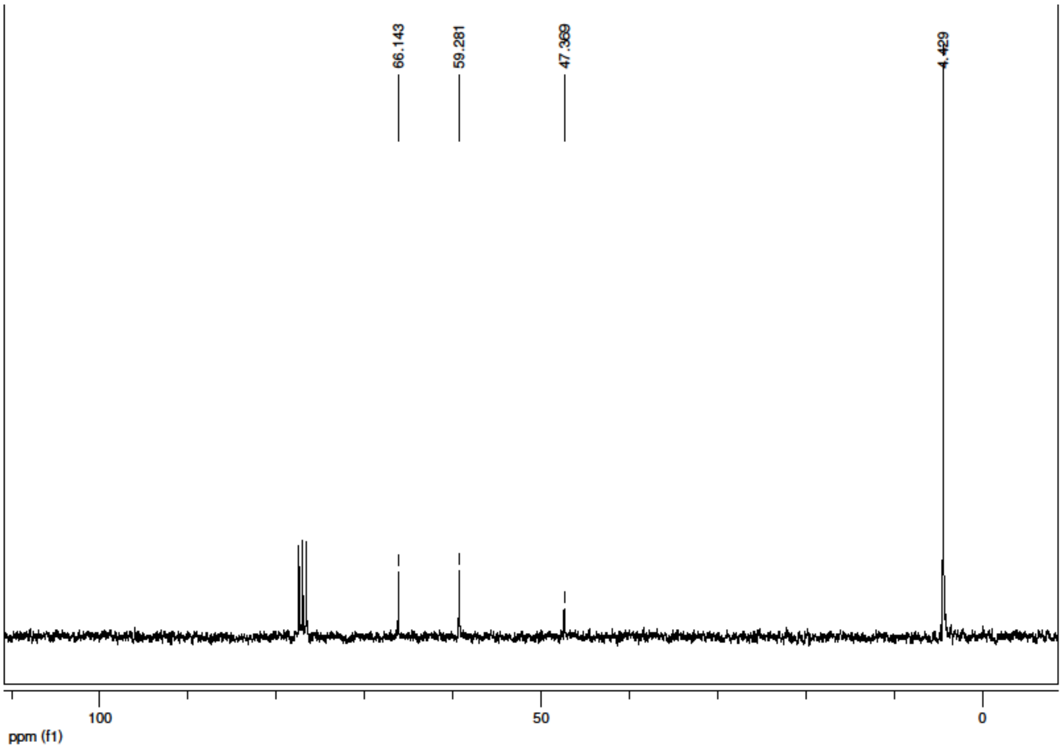

Figure S14. ^{13}C NMR spectrum of **8a** in $CDCl_3$.

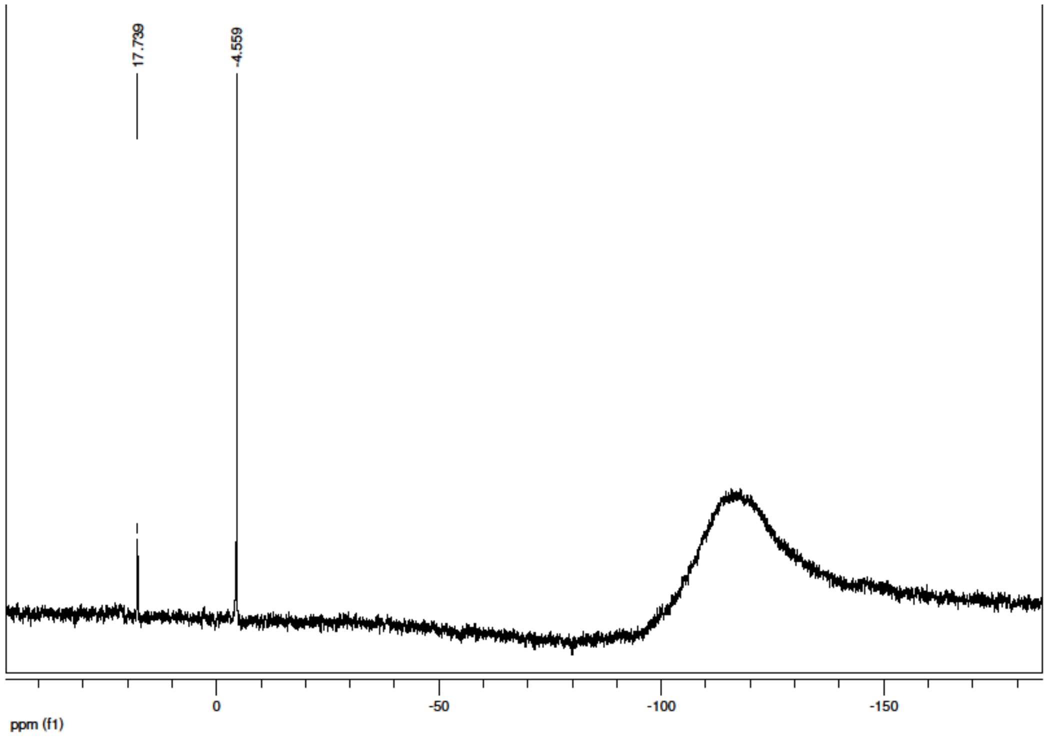

Figure S15. ^{29}Si inversed gated NMR spectrum of **8a** in $CDCl_3$.

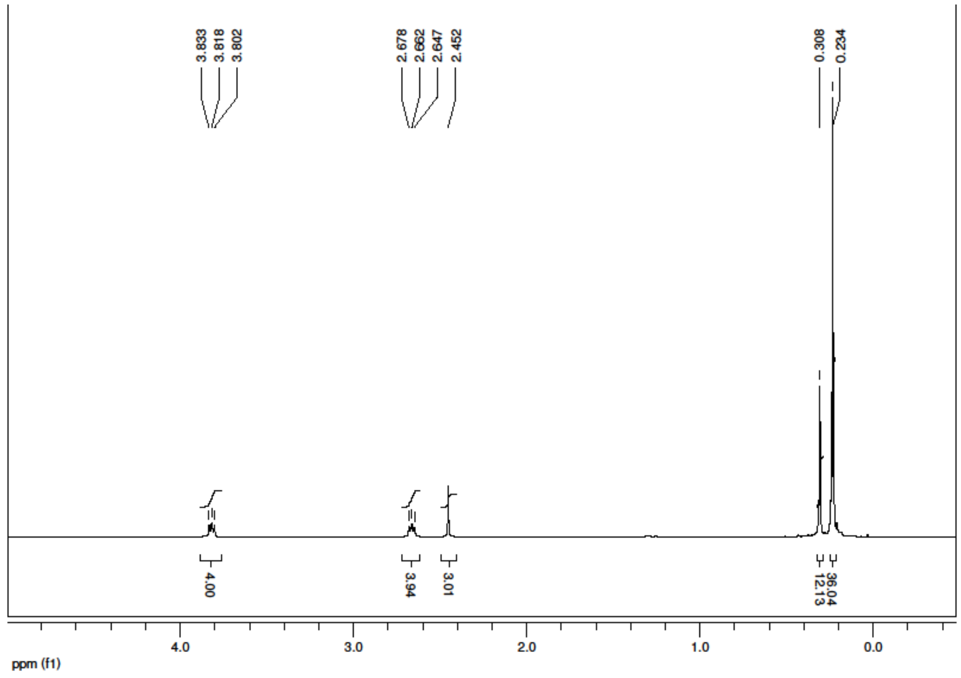

Figure S16. ^{1}H NMR spectrum of **9** in $CDCl_3$.

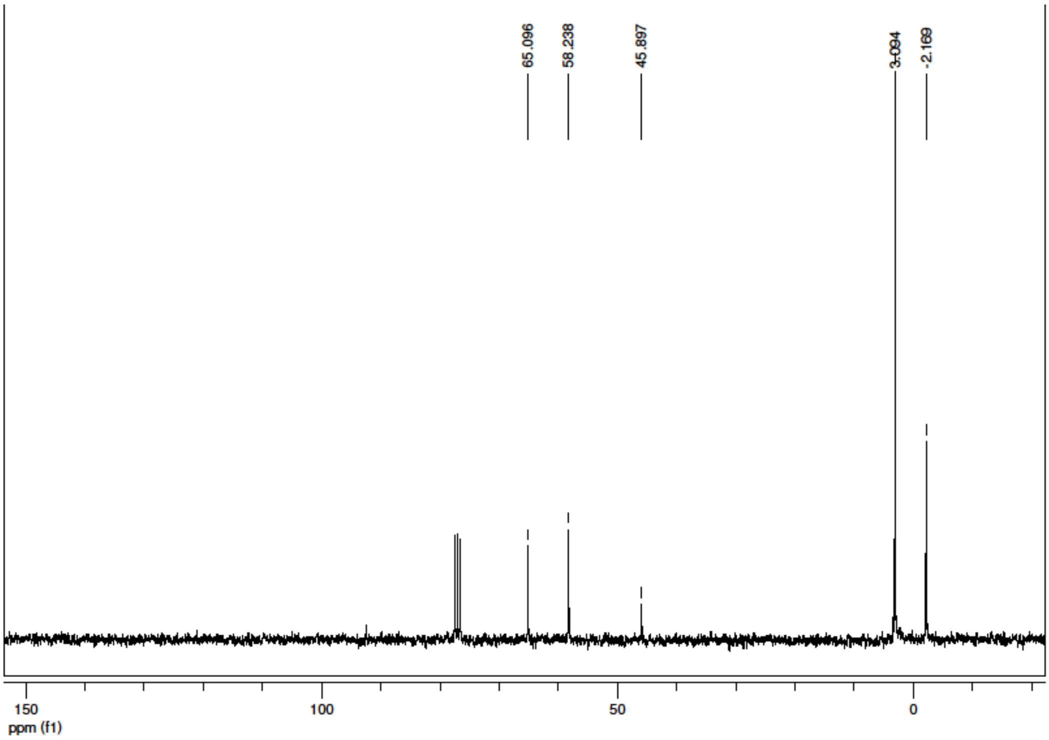

Figure S17. ^{13}C NMR spectrum of **9** in $CDCl_3$.

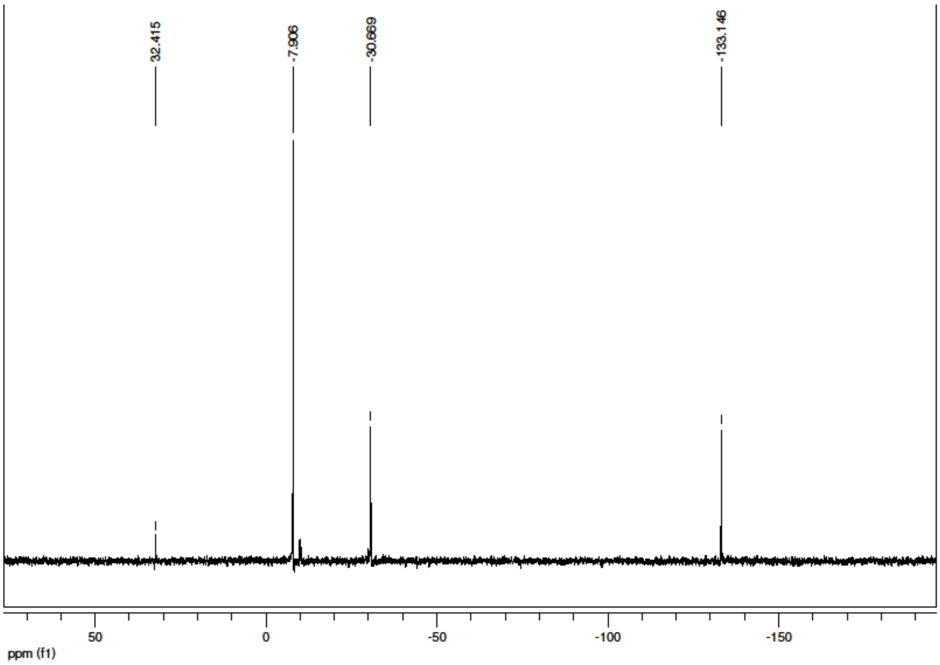

Figure S18. ^{29}Si INEPT NMR spectrum of **9** in $CDCl_3$.

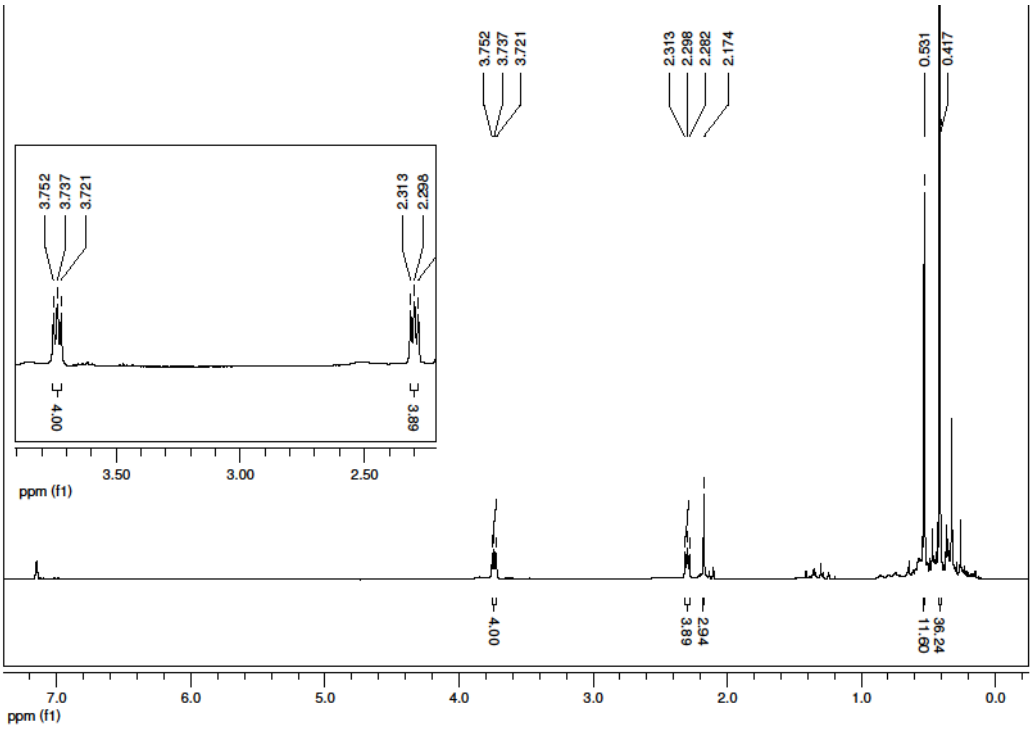

Figure S19. ^{1}H NMR spectrum of **10** in C_6D_6.

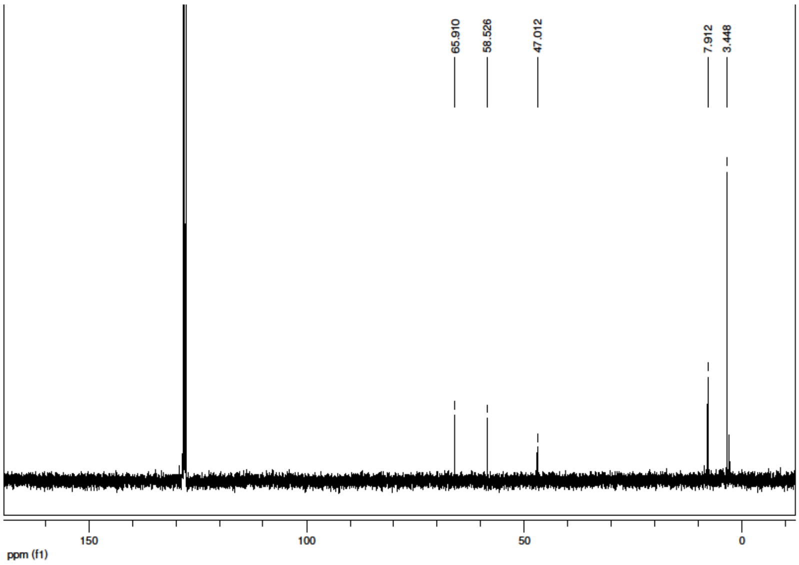

Figure S20. ^{13}C NMR spectrum of **10** in C_6D_6.

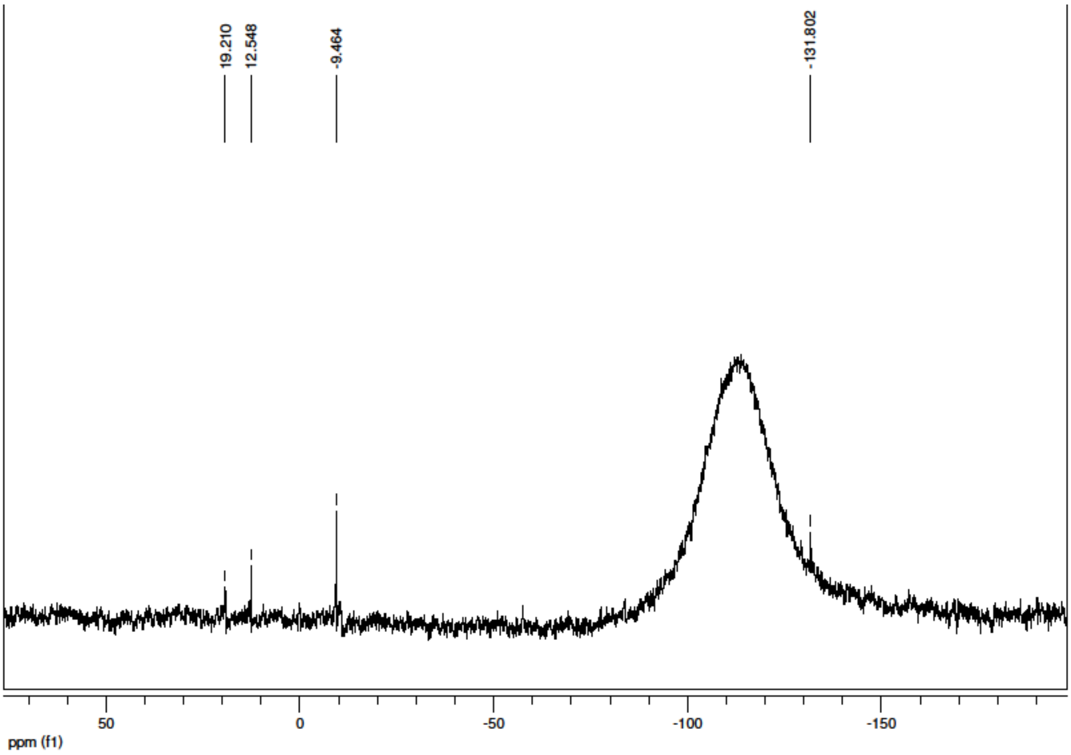

Figure S21. ^{29}Si inversed gated NMR spectrum of **10** in C_6D_6.

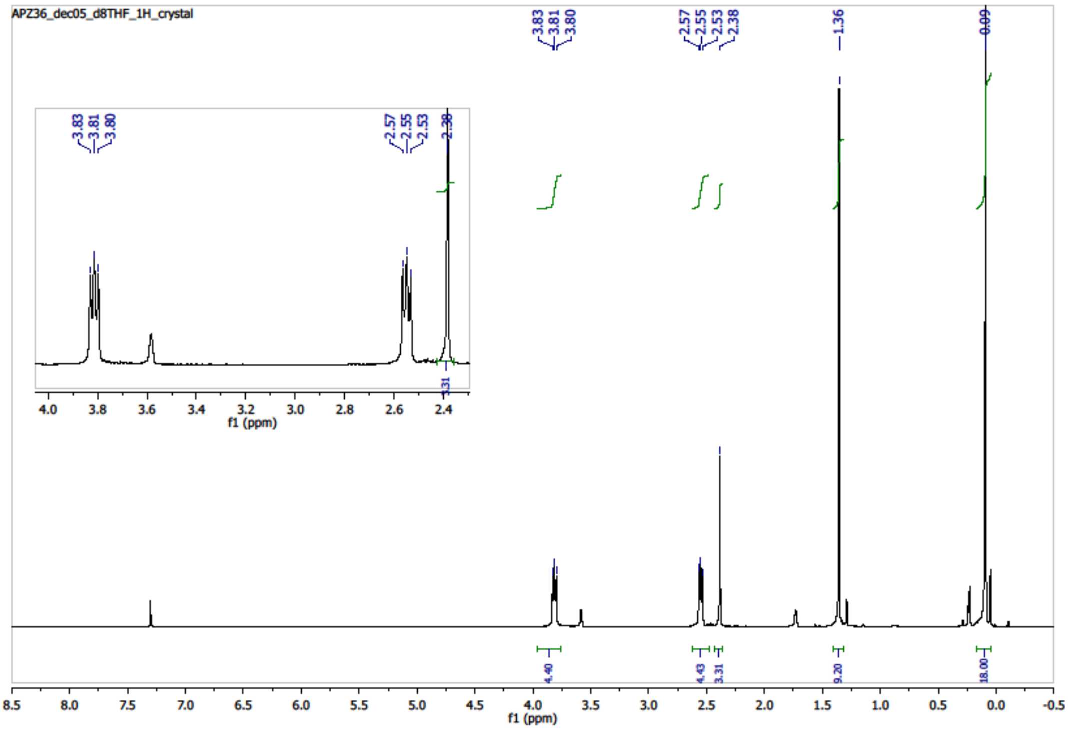

Figure S22. ^1H NMR spectrum of **12** in THF-d_8.

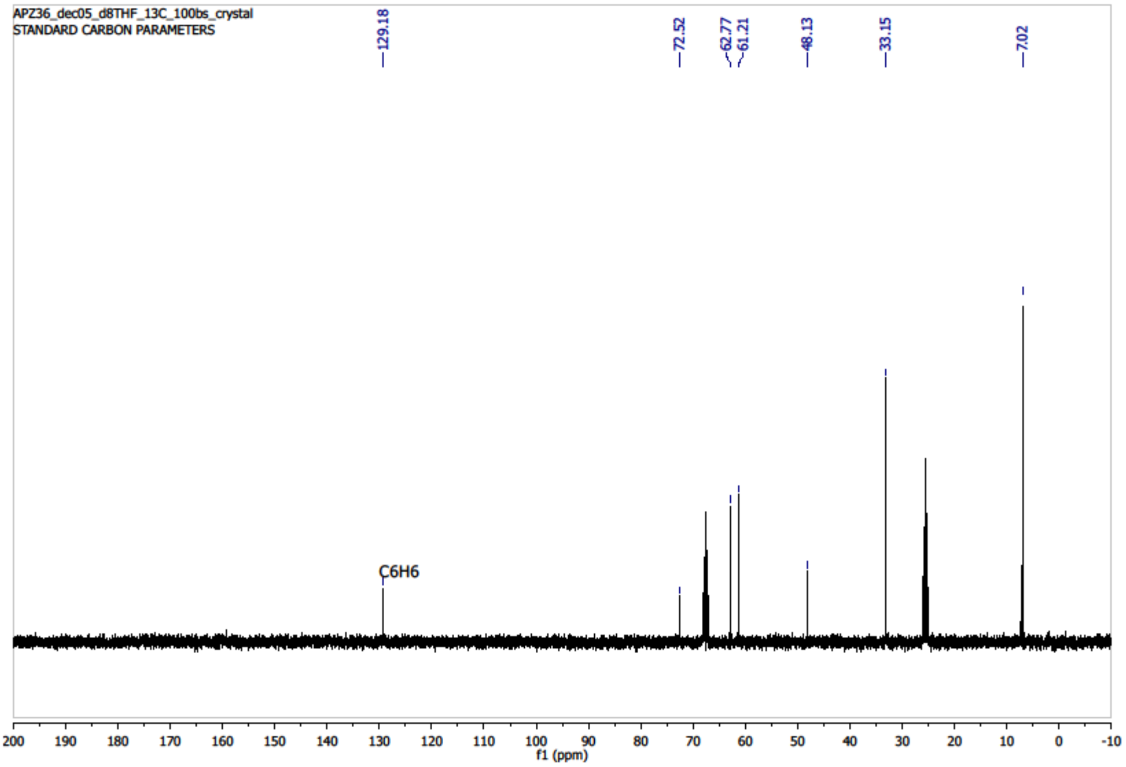

Figure S23. ^{13}C NMR spectrum of **12** in THF-d_8.

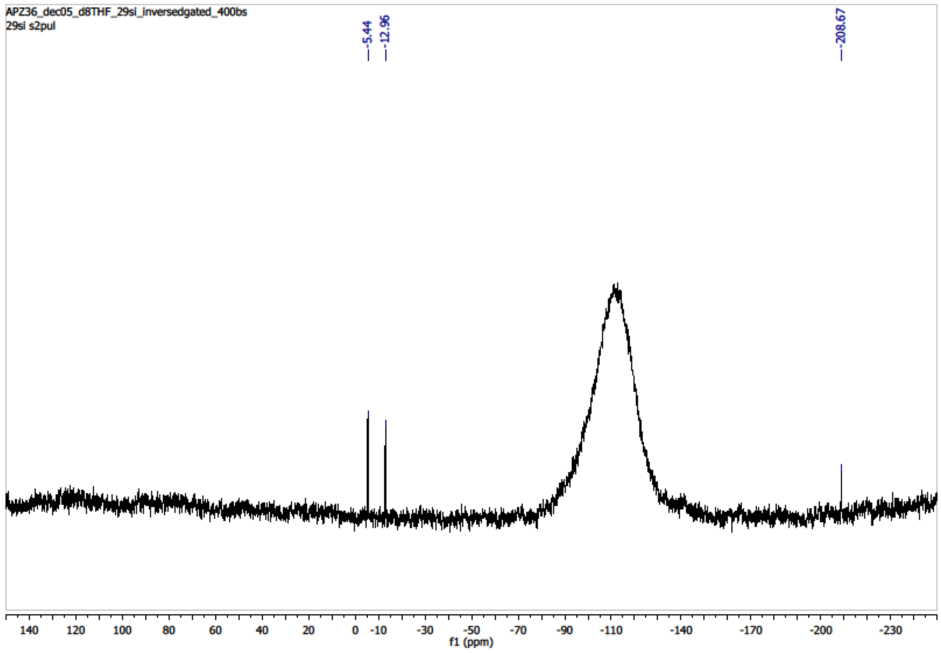

Figure S24. ^{29}Si inversed gated NMR spectrum of **12** in THF-d_8..

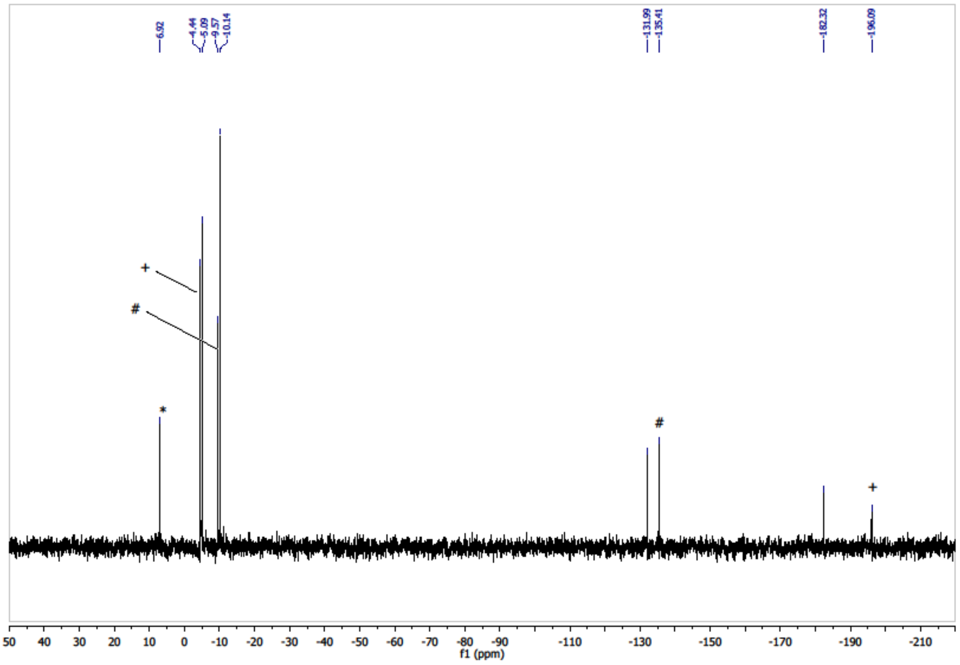

Figure S25. ^{29}Si INEPT NMR of **13** (in the reaction mixture together with Me_3SiO^tBu (*), $(Me_3Si)_4Si$ (#), and $(Me_3Si)_3SiK$ (+)) in DME.

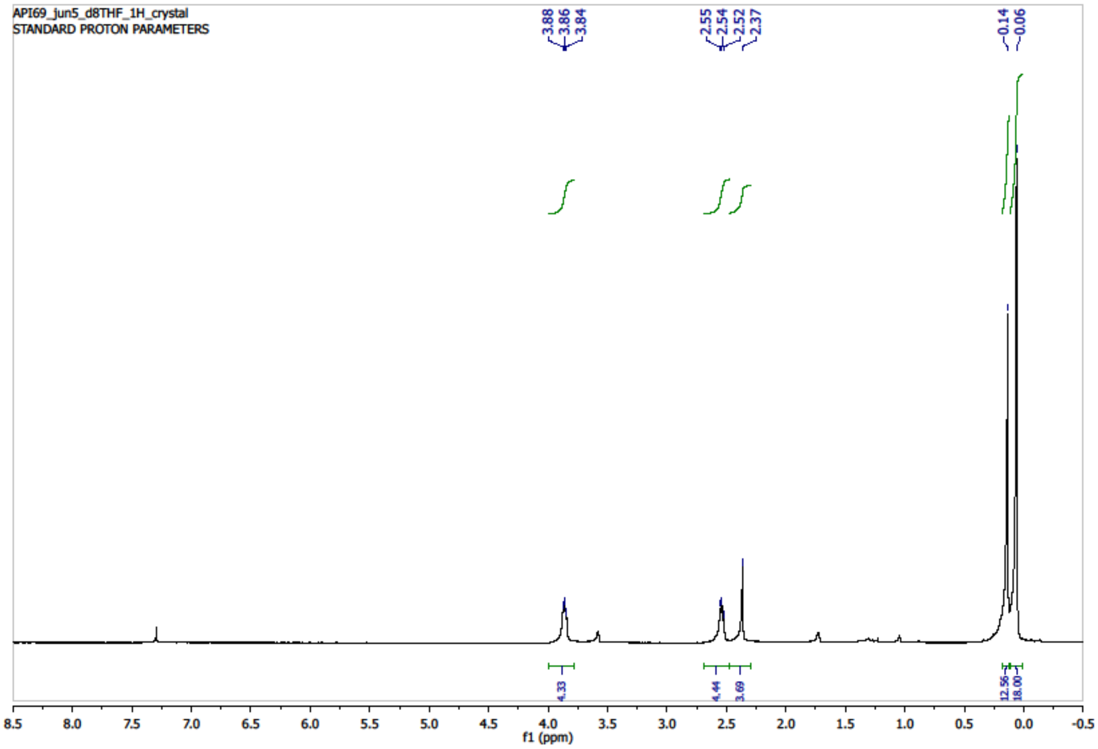

Figure S26. ^{1}H NMR spectrum of **14** in THF-d_8.

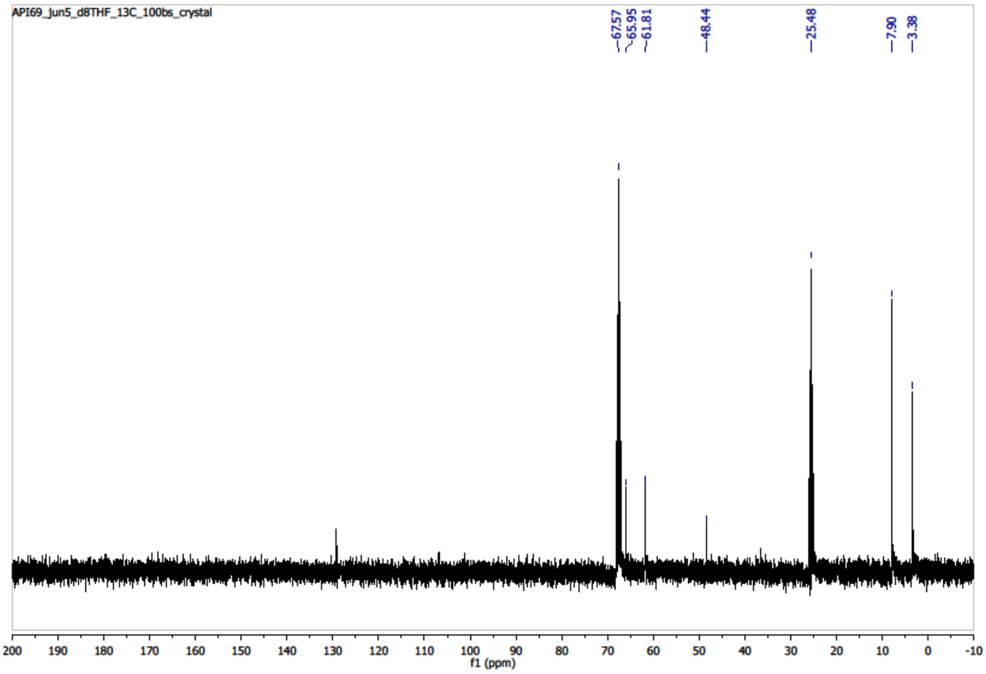

Figure S27. ^{13}C NMR spectrum of **14** in THF-d_8.

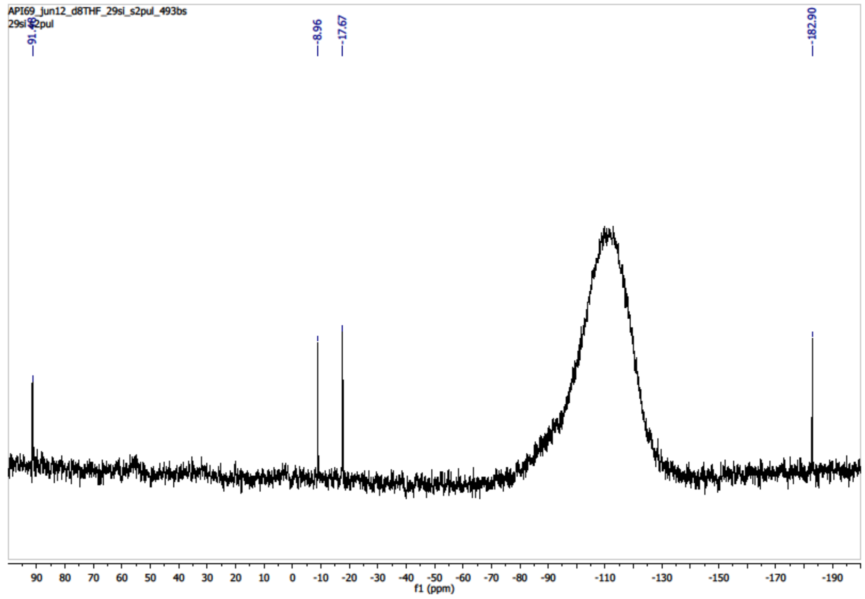

Figure S28. ^{29}Si inversed gated NMR spectrum of **14** in THF-d_8.

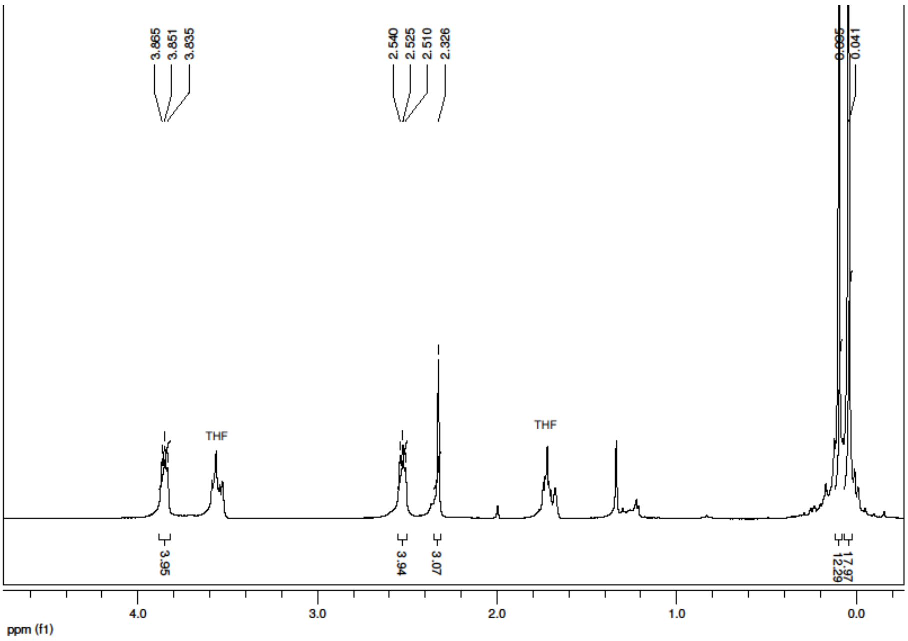

Figure S29. ^{1}H NMR spectrum of **15** in THF-d_8.

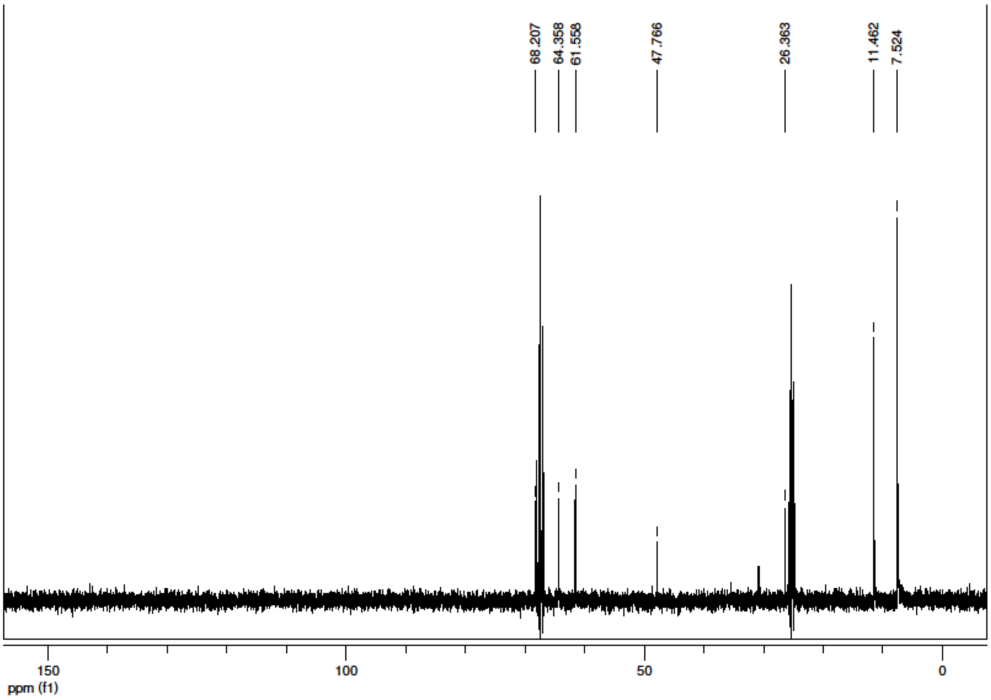

Figure S30. ^{13}C NMR spectrum of **15** in THF-d_8.

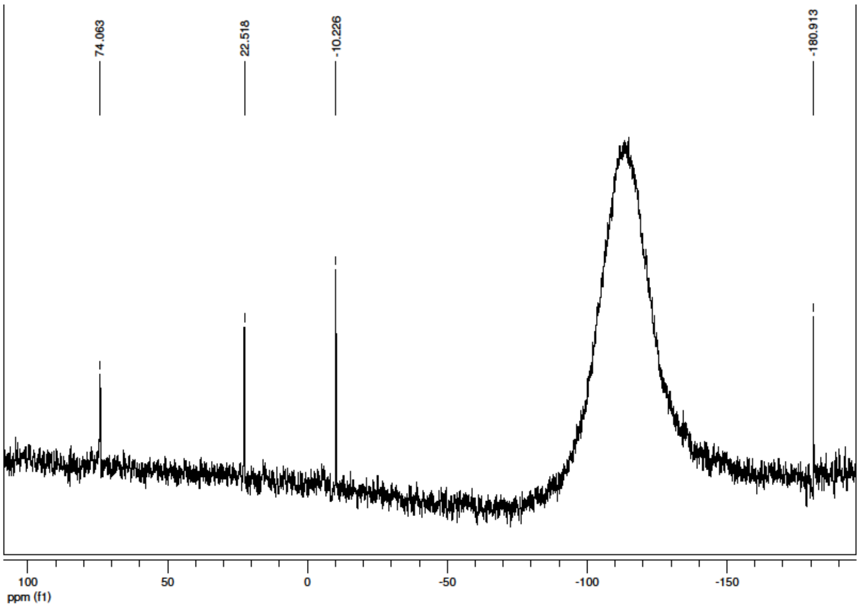

Figure S31. ^{29}Si inversed gated NMR spectrum of **15** in THF-d_8.
